# Recent Advances in Studying Toll-like Receptors with the Use of Computational Methods

**DOI:** 10.1021/acs.jcim.3c00419

**Published:** 2023-06-07

**Authors:** Maria Bzówka, Weronika Bagrowska, Artur Góra

**Affiliations:** † Tunneling Group, Biotechnology Centre, Silesian University of Technology, 44-100 Gliwice, Poland; ‡ Department of Organic Chemistry, Bioorganic Chemistry and Biotechnology, Faculty of Chemistry, Silesian University of Technology, 44-100 Gliwice, Poland

**Keywords:** immune response, pattern recognition receptors, small-molecule modulators, Toll-like receptors, vaccine design, protein−ligand interactions, protein−protein interactions signaling

## Abstract

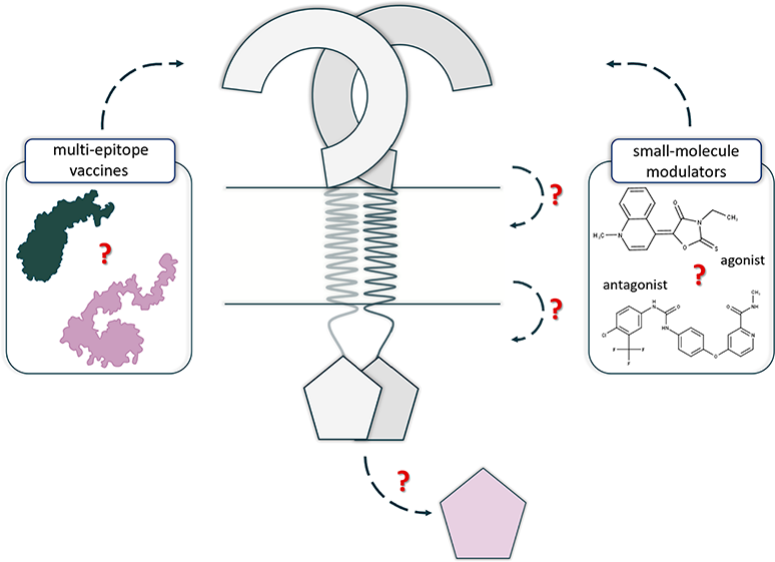

Toll-like receptors (TLRs) are transmembrane proteins that recognize various molecular patterns and activate signaling that triggers the immune response. In this review, our goal is to summarize how, in recent years, various computational solutions have contributed to a better understanding of TLRs, regarding both their function and mechanism of action. We update the recent information about small-molecule modulators and expanded the topic toward next-generation vaccine design, as well as studies of the dynamic nature of TLRs. Also, we underline problems that remain unsolved.

## Introduction

**Figure 1:**
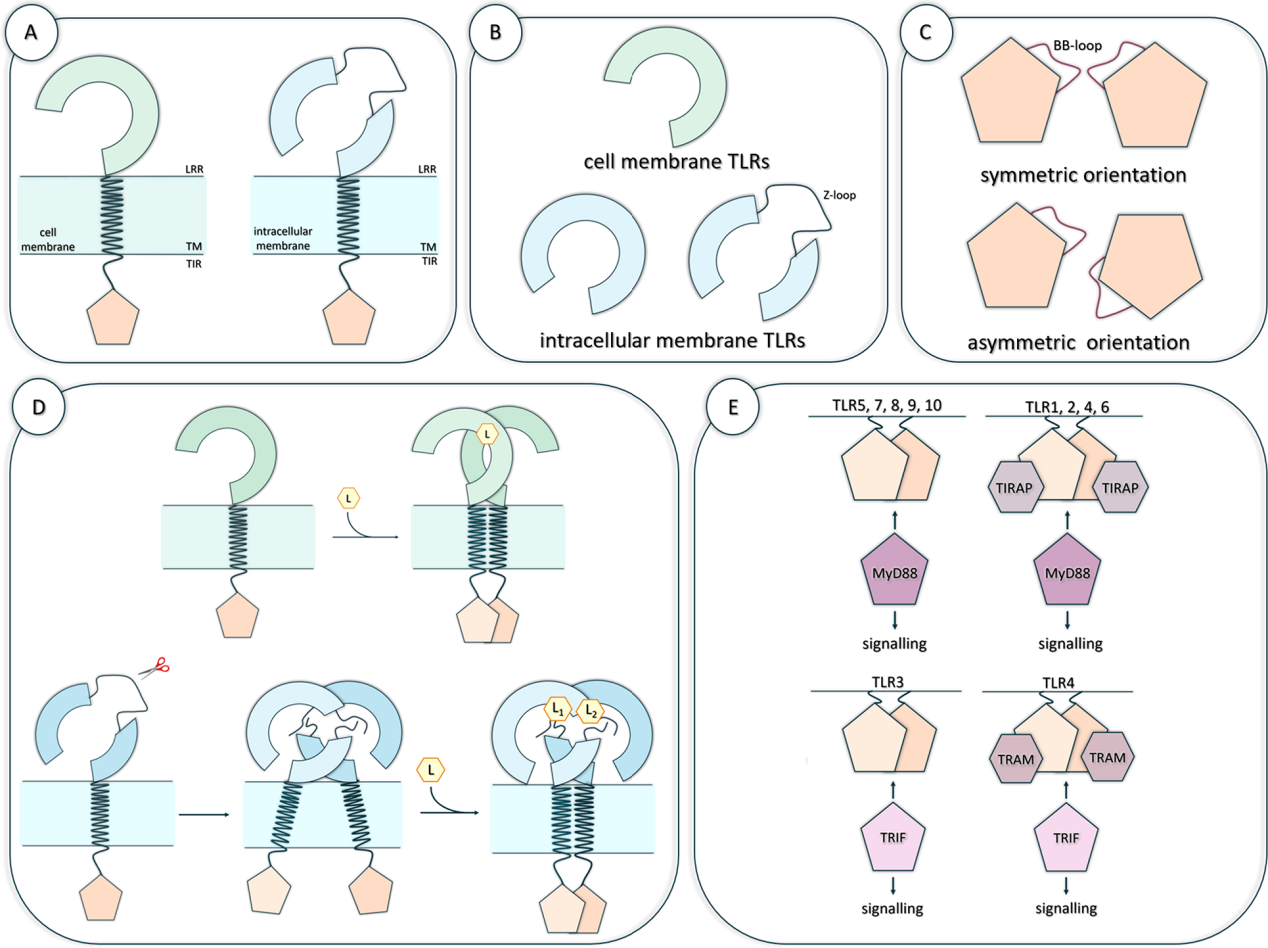
Structural organization and potential Toll-like receptors (TLRs) mechanism of action. (A) The general structure of the TLRs’ monomers. (B) Differences in the TLRs’ LRR domains between the cell membrane and intracellular membrane TLRs. (C) Various orientations (symmetric and asymmetric) of the TIR domain subunits in the TLRs’ TIR dimer. (D) Potential mechanisms of the TLRs activation. The upper panel shows the mechanisms of the cell membrane TLRs activation, while the lower panel presents the mechanisms of the intracellular membrane TLRs containing a Z-loop. (L) indicates the ligand, while the scissors symbol indicates the proteolytic cleavage of the Z-loop. (E) Binding of the adaptor proteins, MyD88 and TRIF, to the respective TLRs’ TIR dimer.

Toll-like receptors (TLRs) represent one of the families of pattern recognition receptors (PRRs) and are an important part of the innate immune system.^1,2^ They are able to recognize various molecular patterns (MPs) in the host organism: damage/danger-, microbial/microbe-, pathogen- or xenobiotic-associated (DAMPs, MAMPs, PAMPs, or XAMPs, respectively).^3−5^ Recognition of those MPs activates downstream signaling cascades that lead to the induction of the innate immune system.^6−8^ In humans, TLRs comprise ten functional members (TLR1–10) that share similar domain organization: an N-terminal domain containing the leucine-rich repeats (LRRs), a single transmembrane helix (TM), and a C-terminal cytoplasmic Toll-interleukin-1 receptor (TIR) domain (Figure 1A). TLR7–9 possess an additional long-inserted loop region (so-called Z-loop) in their LRR domain (Figure 1B) that needs to be cleaved proteolytically. The LRR domain is responsible for ligand recognition, while the TIR domain interacts with adaptor proteins and is responsible for initiating signal transduction. A characteristic feature of the TIR domain in all TLRs is the conserved and functionally important BB-loop (Figure 1C). TLRs are expressed either on the cell surface (TLR1, 2, 4, 5, 6, 10; occasionally TLR7) or in the various intracellular compartments (TLR3, 7, 8, 9; occasionally TLR4). The location of TLRs determines the spectrum of ligands they are able to recognize. For instance, TLRs expressed on the cell surface primarily recognize microbial membranes and/or components of the cell wall, while intracellular TLRs principally recognize nucleic acids.^9−11^ The full list of the recognized ligands is much larger and has been discussed in several papers.^11−14^ The binding of ligands to a TLR either induces the formation of a receptor dimer or changes the conformation of a preexisting dimer (Figure 1D), which subsequently allows adaptor proteins to bind and trigger an immune response.^15^ TLRs can recruit various adaptor proteins; however, myeloid differentiation primary-response protein 88 (MyD88) and TIR domain-containing adaptor protein inducing interferon-β (TRIF) are the most important ones. Two distinct signaling pathways used by TLRs start from them—MyD88-dependent and TRIF-dependent pathways. In general, the MyD88-dependent pathway is utilized by all TLRs, except TLR3, and leads to the production of various proinflammatory cytokines. The TRIF-dependent pathway is utilized by TLR3 and 4 and is associated with the stimulation of type-I interferon^16−19^ (Figure 1E).

Toll-like receptors are a potential therapeutic target in various diseases and conditions. Thus, searching for and designing compounds that can act as agonists or antagonists is the objective of many studies. The distinction between agonists and antagonists for TLRs is crucial since they are used to treat different conditions. For instance, TLR agonists have been developed to treat allergies, asthma, different types of cancer, and chronic infections by upregulating the innate immune system. Moreover, since TLRs induce the response of the body’s defenses, they are also promising targets for designing vaccines. On the other hand, TLR antagonists have been used to treat many inflammatory conditions such as acute/chronic inflammation, sepsis, chronic obstructive pulmonary diseases, cardiovascular diseases, neuropathic and chronic pain, and various autoimmune diseases.^20−23^

In recent years, multiple studies have been published, in which TLRs were the main object of research. Particular studies were focused on the following aspects regarding Toll-like receptors: their structure, ligand recognition, signal transduction, and modulator design. Some of these works were done with the use of *in silico* methods. Due to the increase in the use of computational techniques, it was our goal to summarize how various *in silico* solutions have contributed to a better understanding of TLRs. More than five years have passed since the last published reviews on this topic,^24−26^ and we decided to gather the latest relevant results in this paper. We summarized the research conducted so far, while also emphasizing in which areas we still lack knowledge or solutions. In this work, we focused exclusively on research on human Toll-like receptors (hTLRs).

## Available Structures of TLRs

The first solved structures of hTLRs—TIR domains of TLR1 and TLR2—have been available since 2000,^27^ while the LRR domain of TLR3 has been available since 2005.^28,29^ In the case of the TM helix, the first structures were elucidated in 2014 as the result of an NMR experiment.^30^ The vast majority of available structures have been deposited in the Protein Data Bank (PDB)^31^ in the past decade (Supplementary Table S1). However, almost all are single domains of TLRs. Obtaining full-length structures of TLRs remains a challenge. So far, only the LRR and TM domains of TLR3 and TLR7 have been determined together as a result of the Cryo-EM experiment.^32^ Furthermore, there is a large disproportion in the number of structures between the individual members of the TLRs family. The biggest number of structures has been deposited for the LRR domain of TLR8. In contrast, other TLRs have very few (or none) representative structures of their particular domains. Investigation of the available structures revealed that a part of them miss a number of residues, which worsens their overall quality. Moreover, some deposited LRR domains of TLR1, TLR2, and TLR4 are hybrids of human TLR with hagfish variable lymphocyte receptor B. Those factors make not only the structural analysis but also studies on ligand binding, receptor activation, signal transduction, and modulator design not trivial. An interesting combination of computational and experimental approaches was applied for the identification and understanding of the Zn binding to the TIR domain.^33^ Lushpa et al. proposed a hypothesis in which Zn^2+^ ions can bind to the TLR1 TIR domain BB-loop and stabilize the conformation of the domain, which interact with TLR2 TIR domain or adaptor proteins. With the use of the NMR experiment, the authors confirmed that the computationally obtained two modes correspond to distinct conformations of the BB-loop and that Zn binding may affect the dynamics and conformational landscape of the BB-loop in the TIR domain. Another example of the use of solution NMR combined with computational simulations has been recently published.^34^ Kornilov et al. contributed to resolving one of the major “blank spots” in the structure of TLRs, which was the conformation of their transmembrane domains and cytoplasmic juxtamembrane (JM) regions. The authors identified a new structural element, the cytoplasmic hydrophobic JM α-helix, which plays an important role in TLR activation and connects the transmembrane and cytoplasmic parts of the receptor. As they pointed out, the role of the JM region is more complicated than that of a TM-TIR linker and should not be underestimated in further studies.

Recently, we have entered an era where we have gained relatively straightforward access to the prediction of structures. Models of full-length TLRs structures in their monomeric form can be found in the repository of the AlphaFold Protein Structure Database.^35,36^ Still, one needs to remember that in the case of the predicted structures, they need to be carefully assessed in terms of their quality and usability.

## Computational Studies on TLRs

Review articles on computational methods applied in the Toll-like receptors research published before 2017 cover mostly the topics related to designing small-molecule modulators of TLRs.^24−26^ For instance, Murgueitio et al.^24^ described three main application areas of computational methods to the discovery of TLR modulators: (i) exploration of the structure and function of the receptor, (ii) analysis of receptor–ligand interactions, and (iii) rational design of novel TLR agonists and antagonists by virtual screening (VS). In another work, Pérez-Regidor et al.^25^ focused almost exclusively on the search for novel chemical modulators for TLRs employing VS techniques. Not only did the authors provide information about the available results for five members of the TLRs family—TLR2, 3, 4, 7, and 8—but also they described the available information about the databases, protocols, and techniques used in virtual screening. In their review, Billod et al.^26^ focused on TLR4 exclusively and summarized the following aspects: a perspective of the TLR4/MD2/ligand recognition and dimerization, mutant studies, binding mode modulators analysis, and VS strategies for various types of modulators. In 2020 Wang et al. published an article aimed at the progress in developing TLR signaling pathway modulators.^37^ They mainly focused on the results provided by Yin and Wang laboratories and discussed the identification and characterization of new chemical entities, their modes of action, and further applications. For works that used computational methods, they provided such information in the paper. Based on the results summarized in those reviews, it is clear that almost all the studies focused on finding small-molecule modulators for the LRR domain of the TLRs. As rightly noted by Wang et al.,^37^ TM domains are usually considered “undruggable” and TIR domains among TLRs are highly conserved, which is why most modulators are designed to target the LRR domain of TLRs.

Below, we summarized substantial studies that have been published in recent years in which computational methods have been employed. First, we gathered the recent works that focused primarily on designing modulators for TLRs. In particular, we focused on two types of modulators: small-molecule and vaccine components. While small-molecule compounds have been extensively studied, vaccine components have not been reviewed in detail. Second, we reviewed studies principally focused on the investigation of the dynamic nature of TLRs, which is crucial for understanding their function and mechanism of action.

## Modulators of TLRs

The search for new chemical entities as potential TLRs modulators is an ongoing process, especially because relatively few compounds with therapeutic potential have been tested in clinical trials. Additionally, the use of a strategy involving the TLRs as a driving force for the design of next-generation vaccines has become increasingly popular recently. Since different types of modulators (small-molecule or part of the vaccines, e.g., epitopes) require various methods and techniques for their identification, we reviewed both classes separately.

### Novel Potential Small-Molecule Agents

**Figure 2:**
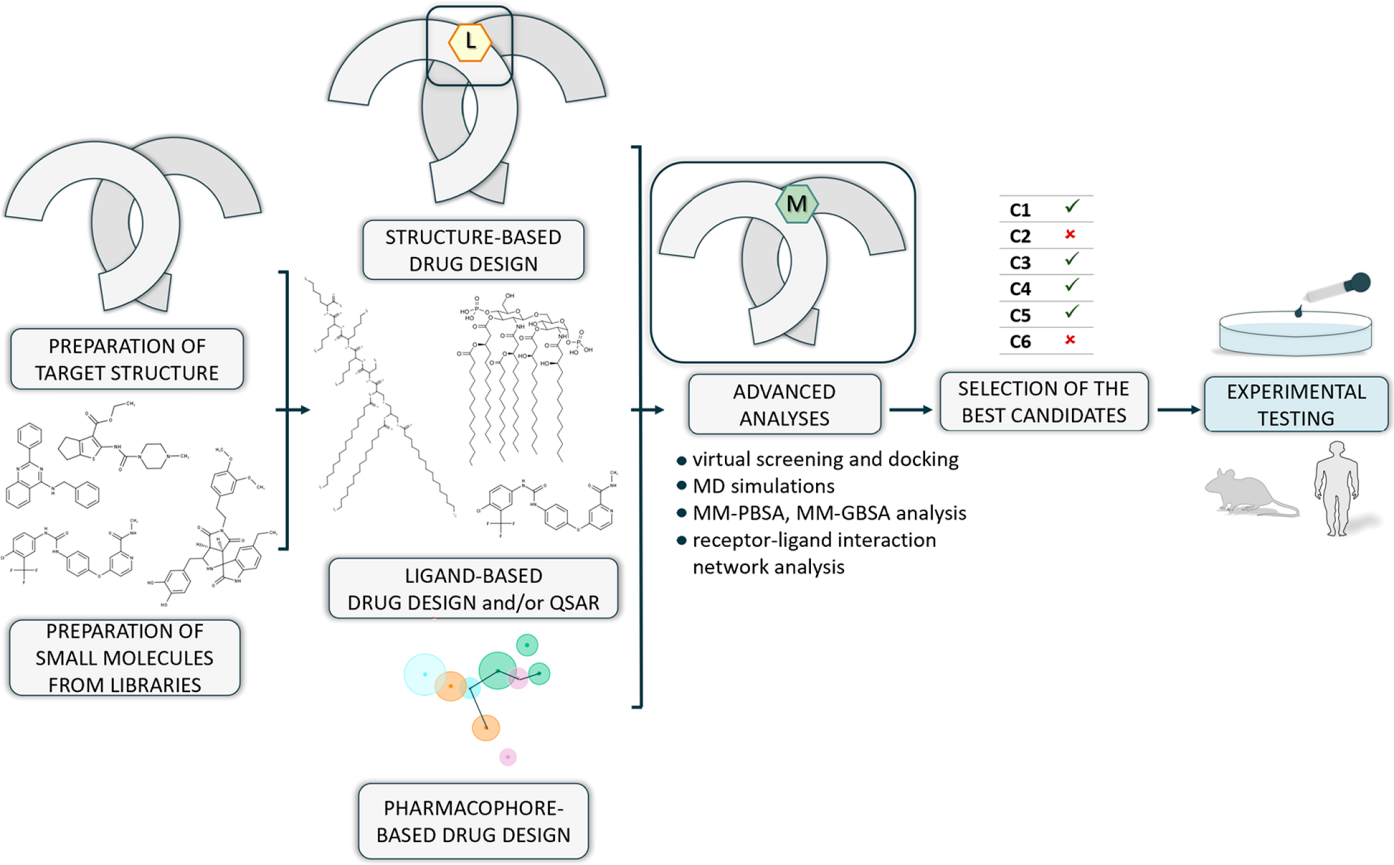
A general protocol for small-molecule modulators design targeting the LRR domain of TLRs. The subunits of the LRR domain are colored gray, indicating both TLRs located in the cell membrane and TLRs located in the intracellular compartments. (L) indicates the location of the ligand binding site, while (M) points out the designed modulator and (C) the selected candidate(s).

The general protocol used for the search for novel small-molecule TLRs modulators has remained the same in most of the studies conducted so far. It consists of the following steps: (i) preparation of the target structure, (ii) preparation of small molecules from available libraries, (iii) structure-, ligand, and/or pharmacophore-based virtual screening combined with molecular docking, (iv) selection of best candidates, (v) experimental testing, and (vi) identification of potential drug candidates. Before the selection of the best candidates, more advanced computational methods are sometimes used, e.g., molecular dynamics (MD) simulations, MM-PBSA, MM-GBSA binding free energy analysis, combined with receptor–ligand interaction network analysis (Figure 2). By applying those advanced methods it is possible to gain better insight into the molecular basis of ligand recognition. Usually, all-atom MD simulations of the receptor–ligand complex are performed.

For VS, scientists have various commercial, public, or in-house databases at their disposal. Many groups have concentrated on modifying the previously identified small-molecule compounds or mimicking the native ligands within known binding sites. Nevertheless, there are also examples revealing novel chemical classes of potential modulators. Studies conducted so far are still mainly focused on targeting the LRR domain of TLRs. There has been no noticeable progress in the design of modulators for the TIR domain.

Many recent studies have been carried out on TLR2. For instance, Murgueitio et al.^38^ performed a shape- and feature-based similarity VS (with the use of ROCS software) to screen some commercially available databases (LifeChemicals, Maybridge, Chembridge, Enamine HTS Collection, Asinex, and Specs). For the similarity search, they used the previously discovered TLR modulators from Guan et al.^39^ and Liang et al.^40^ The authors tested selected hits, and four (AG1-AG4) were found to synergistically increase the nuclear factor kappa-light-chain-enhancer of activated B cells (NF-κB) activation induced by the known lipopeptide ligand Pam_3_CSK_4_. Further studies indicated that the tested compounds could act as ago-allosteric modulators of TLR2. To investigate the binding modes of the identified compounds, the authors run docking calculations (GOLD Suite), using the crystal structure of the human TLR2/1 heterodimer in complex with Pam_3_CSK_4_ (PDB ID: 2Z7X). They inspected the docking poses in LigandScout and identified a putative binding site in the vicinity of the Pam_3_CSK_4_ binding site which is formed during the heterodimerization process. Information about the characterized interacting residues can be found in Supplementary Table S2. For other compounds described in the following parts of this section, details on the identified interacting amino acids (if available in the cited publications) are also provided in Supplementary Table S2.

Durai et al.^41^ used receptor–ligand- and ligand-based VS to prepare the pharmacophore models and to screen in-house libraries comprising nearly seven million compounds. They focused on the nonpeptide TLR2 antagonists, distinct from several known inhibitors with fatty acid chains. For the receptor–ligand-based model, the authors prepared the protein–lipopeptide complex (PDB ID: 2Z7X),^42^ while for the ligand-based model, they selected compounds from Guan et al.^39^ They used the Receptor–ligand Pharmacophore Generation and Common Feature Pharmacophoric Generation protocols (Discovery Studio Visualizer Software, 4.0), respectively. The next steps involved screening the compounds that mapped to the pharmacophore features and filtering them using Lipinski and Veber rules, as well as ADMET properties. The authors evaluated the best hits for their ability to bind directly to the lipopeptide binding site of the human recombinant TLR2. For that, they performed a two-step molecular docking (CDOCKER and AutoDock Vina) and tested the selected protein–ligand docking complexes by MD simulations combined with MM-PBSA binding free energy calculations (GROMACS with CHARMM27 force fields and SPC216 water model). The authors selected promising TLR2/1 antagonists using surface plasmon resonance experiments and tested their ability to inhibit the synthesis and secretion of IL-8 in human embryonic kidney cells overexpressing TLR2. Two molecules—C11 and C13—displayed both direct binding to TLR2 extracellular domain and reduced Pam_3_CSK_4_-induced IL-8 production. Those antagonists showed no toxic effect in cell viability assays and seemed to have good pharmacological properties. The results supported the possibility that C11 and C13 can disrupt TLR2/1 heterodimerization.

Chen et al.^43^ performed a structure-based VS (Glide) of the ZINC database. Based on the scoring results, including shape, chemical-feature, and drug-like properties, they identified potential agonists targeting the TLR2 heterodimer and modulating the TLR2/1 response. For the most promising candidates, which shared a motif of an amine conjugated with an acid substituent, they tested their activity *in vitro*. The results revealed that two compounds showed a high TLR2 activation effect and that one compound—ZINC6662436 (SMU127)—stimulated the NF-κB and promoted tumor necrosis factor-α in human macrophage and mononuclear cells. Also, the *in vivo* results showed signs of inhibition of breast cancer tumor growth in BABL/c mice. In a later study, Chen et al.^44^ improved the potency of the SMU127 by modifying the ring system, while keeping all other structural features. One of the modified compounds—SMU-C13 possessed the highest TLR2 activity. This compound was docked into the 2Z7X structure (Glide) and evaluated regarding its putative binding. The *in silico* simulation indicated a tight fit into the known binding site of Pam_3_CSK_4_ and TLR2/1. Based on the structure–activity relationship (SAR) results, the authors concluded that the introduced piperidine ring contributed to the increased activity against TLR2.

Grabowski et al.^45^ performed both ligand- and structure-based VS using commercial databases of nearly six million compounds (Asinex, LifeChemicals, Mybridge, Chembridge, Enamine, Otava, Specs, Vitas-M, KeyOrganics, and ChemDiv). The authors selected two well-characterized chemotypes of small-molecule modulators to build their models—(i) m1 proposed in previous work by Murgueitio et al.^46^ and (ii) CU-CPT22 and the other benzotropolones discovered by Yin et al.^47^ For that, they applied a standard protocol for pharmacophore-based screening (LigandScout). For all modeling studies, the authors used a TLR2 monomer from the TLR1-TLR2 heterodimer (PDB ID: 2Z7X). Screening of compounds was followed by their filtering using shape- and feature-based properties. Then, the authors carried out docking (GOLD), rescoring, and visual inspection analyses and selected the best hits for biological testing to confirm their ability to inhibit TLR2-mediated responses. Selected compounds were tested in HEK293-hTLR2 cells, THP-1 macrophages, and peripheral blood mononuclear cells. The most active compound, a pyrogallol derivative named MMG-11 inhibited both TLR2/1 and TLR2/6 signaling. It also showed a higher potency than the previously discovered CU-CPT22. Additionally, in a subsequent paper,^48^ Grabowski et al. confirmed that another potent compound (named compound 8) showed a TLR2 inhibition and additionally reduced TLR7/8 responses. Encouraged by these results, they applied a computationally guided synthesis approach to get an analogue of that compound which showed dual inhibition of TLR2 and 8. For docking studies (GOLD), the authors used the crystal structures with cocrystallized lgands; 5WYZ for TLR8 and 2ZJX for TLR2/1. The authors selected the putative binding modes based on pharmacophore fit rescoring using previously reported TLR2 antagonist MMG-11 and CUCPT9b for TLR8. The results showed that the selected compound 24 is able to simultaneously and selectively target both surface- and endosomal-located TLRs. This compound showed also high efficacy with low cytotoxicity and a noncompetitive antagonist behavior. Also, in another work, Bermudez et al.^49^ explored the chemical space around the pyrogallol-containing antagonists to improve synthetic accessibility and chemical stability.

Boger’s lab proposed a new and potent class of TLRs agonists—diprovocims.^50^ They obtained results from a compound library designed to promote cell surface receptor dimerization. The discovered class of compounds had no structural similarity to any known natural and synthetic TLR agonist, and selected members were confirmed to be active in both human and murine systems. Comprehensive SAR studies improved the potency 800-fold over the screening leads, providing diprovocim-1 and diprovocim-2. The compound 3 of the diprovocim-1 scaffold, later referred as Diprovocim, showed full agonist activity at very low concentrations in human THP-1 cells, being more potent than any other known small-molecule TLR agonist. Later, the basis of TLR2/TLR1 activation by Diprovocim was studied by Su et al.^51^ They combined analysis of the structures of Diprovocim-bound TLR2 homodimer and TLR2/TLR1 in a complex with Pam_3_CSK_4_ with docking, MD simulations (AMBER with ff14SB and GAFF force fields and TIP3P water model), MM-PBSA, MM-GBSA binding free energy and mutagenesis analyses. *In silico* results indicated that binding two Diprovocim molecules to the TLR2/TLR1 heterodimer was slightly less energetically efficient than binding a single molecule. Further analyses revealed that the new modulator interacts with TLR2/TLR1 at the same binding pocket as Pam_3_CSK_4_. However, the observed conformations around the ligand binding sites were different. The Diprovocim-bound TLR2 homodimer showed a larger distance between the C-termini of the TLR2 LRR domain than the Pam_3_CSK_4_-bound TLR2/TLR1 heterodimer, suggesting that the TLR2 homodimer may not be able to activate downstream signaling. The authors noticed the widespread hydrophobic interactions and a hydrogen-bonding network between the receptor and Diprovocim molecules within the ligand binding pocket, while in the Pam_3_CSK_4_-bound receptor complex, such a network was absent. These differences could explain the greater potency of Diprovocim in activating TLR2/TLR1-mediated signaling. The mutagenesis analysis was focused on the identification of which amino acid on TLR1 and TLR2 are important for the binding of Diprovocim and Pam_3_CSK_4_, and all details can be found in the paper of Su et al.^51^

For the TLR4 receptor associated with myeloid differentiation factor 2 (MD2), Mishra and Pathak^52^ aimed at the identification of small-molecule protein–protein inhibitors based on a pharmacophore mapping-based approach. For that, they used information about the generated hot-spot residues (DrugScore^PPI^, KFC2, HotPoint, HotRegion) and their corresponding pharmacophoric features (PocketQuery and ZINCpharmer) on the protein–protein interaction interfaces in the TLR4/MD2 homodimer complex (PDB ID: 3FXI). The authors ran VS with molecular docking (FlexX) and performed extensive post-VS filtration based on ADMET properties, oral bioavailability, and possible side effects—off-targeting and environmental hazard. From selected hits, two (C11 and C15) with the predicted best inhibitory concentration were confirmed to form a stable complex with the target protein during MD simulation analysis (NAMD with CHARMM force field and TIP3P water model). In other studies, Facchini et al.^53^ and Cochet et al.^54^ focused on designing the monosaccharide mimetics of lipid A, which is a known agonist. The authors successfully designed mimetics through docking with MD2 (AutoDock Vina and AutoDock) and confirmed the stability of the modulators by performing MD simulations (AMBER). The compounds were predicted to bind inside the MD2 hydrophobic pocket with favorable predicted binding scores. Subsequently, compounds were synthesized and tested to confirm their ability to bind to MD2 and inhibit LPS-stimulated TLR4 activation.

In general, many known TLR4 modulators are LPS mimetics; however, alternative strategies for finding non-LPS-like modulators have also been applied. A lot of studies focused on the use of opioids and their derivatives. For instance, morphine, cocaine, and methamphetamine (METH) were found to interact with TLR4 to initiate neuroimmune signaling.^55−57^ In their work, Wang et al. performed docking (AutoDock Vina) of METH to the TLR4 receptor (PDB ID: 3VQ2) to investigate how the compound interacts with TLR4/MD2. METH was docked into the dimerization interface of the TLR4/MD-2 complex, and further MD simulation (NAMD, AMBER force field) suggested that the binding of the compound stabilizes the TLR4/MD-2 tetrameric form, which could shift the equilibrium and potentially activate TLR4 signaling as a nonclassic agonist. In another work, Wang et al.^58^ revealed the molecular mechanism of (+)-naltrexone and (+)-naloxone underlying the effects of opioid isomers on TLR4 signaling as the first biased inhibitors of TLR4, which inhibit only the TRIF-dependent signaling with no effects on the MyD88 signaling. These results became the basis for the design of more promising TLR4 antagonists based on known opioids. For instance, Selfridge et al.^59^ designed and synthesized compounds based on (+)-naltrexone and (+)-noroxymorphone. In another study, Zhang et al.^60^ used the previously established protocols to investigate in detail the molecular interactions between (+)-naltrexone, its derivatives, and MD2 of TLR4. Results showed that hydrophobic residues in the MD2 cavity interacted directly with the (+)-naltrexone-based TLR4 antagonists and were essential for ligand binding. Increasing hydrophobicity of the substituted group improved TLR4 antagonistic activity, while charged groups disfavored binding with MD2. MD simulations (NAMD with AMBER03 and GAFF force fields and TIP3P water model) demonstrated that (+)-naltrexone or its derivatives bound to MD2, stabilized its conformation, and blocked TLR4 signaling. The idea of improving naltrexone-based compounds was also developed in later works. An example is the work of Zhang et al.,^61^ who designed bivalent ligands by connecting two naltrexone units through a rigid pyrrole spacer. In a very recent study, Pérez-Regidor et al.^62^ focused on finding non-LPS-like modulators among the approved drugs and drug-like molecules from commercial, public, and in-house libraries of compounds. Based on the structure-, ligand-based VS and docking (FLAP, GLIDE, AutoDock and AutoDock Vina) combined with biological results, the authors presented a common scaffold consisting of two hydrophobic moieties separated by a polar linker. They showed that one large hydrophobic moiety occupies the hydrophobic MD2 cavity, while the second moiety is associated with the same hydrophobic region as one of the lipid A alkyl chains, and the polar linker occupies the entrance to the pocket. Another approach was proposed by Gao et al.^63^ They focused on the computational design of macrocyclic peptides (Rosetta Peptiderive), based on the fragment of MD2 mediating the association of the TLR4/MD-2 complex. The authors synthesized proposed constructs and experimentally evaluated their ability to activate the TLR4 signaling. Application of such approach could potentially overcome the existing problem of targeting protein–protein interaction interfaces which are usually flat and may not be suitable for binding of organic compounds.

An interesting study was performed by Borges et al.^64^ The authors investigated the effect of the natural limonoid gedunin on different TLRs (2, 3, and 4) activation. They performed *in vitro*, *in vivo*, and *in silico* studies. The experimental results confirmed that gedunin is able to impair inflammasome activation, and cytokine production and induce anti-inflammatory factors in macrophages. The docking studies (AutoDock Vina) revealed that the investigated compound can efficiently bind to the TLR2, TLR3, MD2 protein of TLR4, and also to the caspase-1, making gedunin considered a multitarget compound. The authors used the following PDB structures: human caspase-1 (PDB ID: 1RWX), TLR2 (PDB ID: 1O77), and TLR3 (PDB ID: 1ZIW). For both TLR2 and TLR4, gedunin bound within the known ligand binding site, while for TLR3 two distinct binding sites were predicted. The authors pointed out that one of the predicted regions for TLR3 is involved in the dimerization of TLR3 and is considered the dsRNA binding site, thus it might be the most prominent. Still, as pointed out by the authors, further biochemical assays are required to confirm gedunin binding.

For endosomal TLRs—TLR3 and TLR7–9—Talukdar et al.^65^ recently published a perspective paper regarding the structural evolution of their small-molecule agonists and antagonists. They concluded in detail information about structural features around binding sites of both types of modulators, and their evolution and provided information about the development of various chemotypes, e.g., guanosine-, oxoadenine-, 3-deazapurine-, imidazoquinoline-, quinoline-, benzimidazole-, imidazole-, pyridopyrimidine-, pyrrolopyrimidine-, pyrimidine-, quinazoline-, chromene-, benzoxazole-, indole-, triazole-, indazole-, and benzanilide-based.

Here, we wanted to highlight a few studies not included in the above-mentioned publication. One example is the work performed by Gupta et al.^66^ They used the known ligand-based pharmacophore modeling approach to find novel human TLR7 modulators based on the set of TLR7 agonists with confirmed experimental activity. The data set was divided into training and test sets based on criteria such as structural diversity and activity range. The authors created a pharmacophore model (HypoGen algorithm available in 3D-QSAR pharmacophore generation protocol of Discovery Studio) and screened the natural hit compounds from the InterBioScreen Natural product database. They filtered the screened compounds and based on molecular docking (LibDock) and further interaction analyses, they selected the most interesting compound - STOCK1N-65837 (an indoline derivative natural alkaloid). The compound was further validated with MD simulation (GROMACS with GROMOS96 43a1 force field and SPC216 water model). The authors found that STOCK1N-65837 formed hydrogen bond interactions with residues from LRR15 and LRR16 of hTLR7, which was in the good agreement with previous findings that amino acids within that region crucial for ligand binding. The authors underlined that further experimental validation is necessary to confirm the activity of the compound; however, their results already provided a basis for further designing of natural modulators targeting TLRs.

Šribar et al.^67^ used the previously established approach consisting of structure- and pharmacophore-based computational studies (ROCS, GOLD, LigandScout), combined with MD simulations (Desmond), followed up by experimental validation to find novel inhibitors of TLR8. They performed two rounds of VS. The authors used the best hit from the first round of VS and performed its optimization by shape- and chemistry-based screening. Later, they prioritised them according to their diversity and physicochemical properties. Based on that approach, they found a novel pyrimidine scaffold for TLR modulators. Experimental validation of the most promising compounds from the second round of VS revealed their low cytotoxicity, suggesting that they are relevant for further lead optimization.

Recently, Wang et al.^68^ focused on revealing the mechanism of action of known agonists for TLR7 and TLR8 - imidazoquinoline derivatives (Resiquimod (R), Hybrid-2 (H), Gardiquimod (G)). They carried out MD simulations (GROMACS with AMBER ff99SB and GAFF force fields and SPC water model) for both TLR7 and TLR8 apo structures and TLR7 and TLR8 with bound antagonists, followed by the MM-GBSA calculations. Their analysis showed that TLR7-R and TLR7-G complexes formed open conformations during the simulation, while the others were kept in closed conformations. They found that the binding pocket of TLR7 was less flexible than in TLR8, thus the binding of the antagonist was tighter. Moreover, these *in silico* predictions were in agreement with the experimental data.

**Figure 3:**
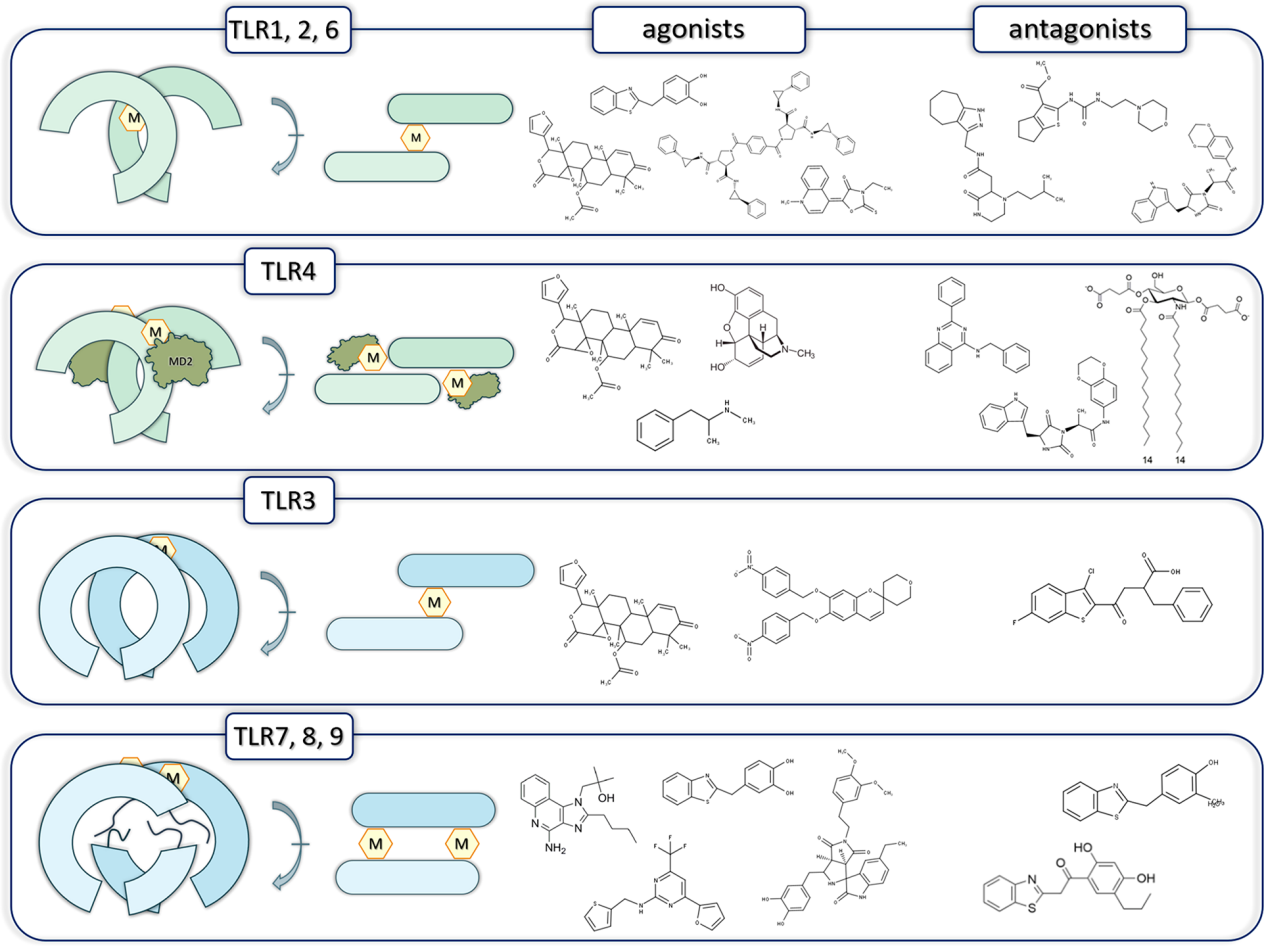
Examples of scaffolds of small-molecule modulators targeting the LRR domain of TLRs. The left panel shows the approximate location of small-molecule modulators (M) with respect to the LRR subunits of the TLR dimers described in this review. Agonists are presented on the middle panel, while antagonists are on the right panel. TLR4 was shown with the associated myeloid differentiation factor 2 (MD2). Please note that one of the agonists’ scaffolds is shown for more than one TLRs. This indicates the possibility of targeting both surface- and endosomal-located TLRs by a given modulator.

In Figure 3 we presented examples of scaffolds of both agonists and antagonists targeting the LRR domain of TLRs proposed in reviewed publications. Also, we showed the localization of the designed small-molecule modulators in relation to the subunits of the TLRs. In Supplementary Table S2 we gathered the structures of all the best hits from the reviewed research papers, as well as the information about the interacting amino acids (if available).

As can be seen from the above-mentioned studies, many groups used the information from the previously designed modulators either for introducing some modifications aimed at increasing their activity or for obtaining models for VS and further studies. In the reviewed papers we encountered both the strategy to design modulators structurally similar to known ligands and compounds with a completely different structure. Interestingly, the targeting sites remain the same, which highlights the challenges in the reconstruction of TLRs structure and difficulties with the identification of other potential binding sites which could affect TLRs function. We could also notice that some of the proposed modulators were able to influence the signaling pathways in various TLRs. Nevertheless, the molecular bases of their selectivity have not been thoroughly examined. Therefore, one needs to keep in mind that we still need in-depth studies revealing the differences in the mechanism of action in relation to different receptors. We believe that in the coming years, more groups will include analyses related to potential off-targeting effects, as well as that there will be an increase in interest in the screening of natural compounds databases for proposing novel small-molecule modulators. Regarding methods, we are expecting an increased contribution of AI-supported screening, especially in ligand-based screening.

### Next-Generation Vaccines

**Figure 4:**
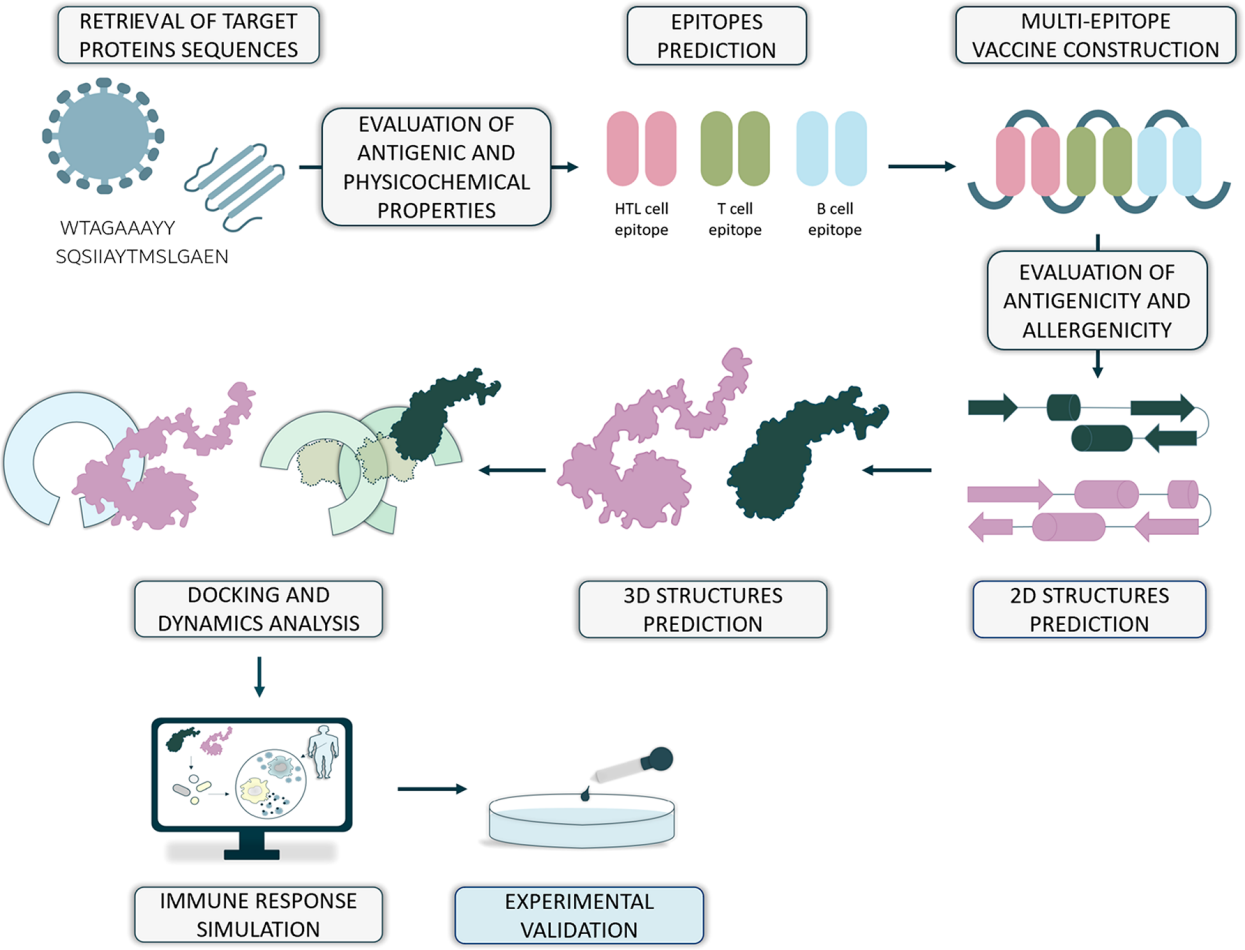
General protocol for next-generation multiepitope vaccine design. The ability of binding different epitopes (shown as dark green and pink shapes, respectively) to LRR subunits of the TLRs located both in the cell membrane (light green) and in the intracellular membrane (light blue) has been shown.

Subunit vaccines are considered one of the next-generation vaccines. They consist of pieces of a pathogen, instead of the whole organism. Evidently, this also means they do not contain any live pathogen and thus show significantly lower immunogenicity. The immunogenicity of the subunit vaccines can be improved by several factors, e.g., addition of adjuvants, choice of different delivery systems, usage of multiple antigens or epitopes, and optimization of vaccine dosage. TLRs are excellent targets for such multiepitope vaccines to provide a signal to induce an effective immune response that in turn leads to long-lasting protection.^23,69,70^ The protocol used for the search for multiepitope modulators is substantially different from the one used for small-molecule modulators. The general protocol consists of multiple steps: (i) retrieval of target proteins sequences, (ii) evaluation of antigenic and physicochemical properties of the target proteins, (iii) epitopes prediction, (iv) multiepitope vaccine construction, (v) evaluation of antigenicity and allergenicity of the vaccine combined with the exploration of the physicochemical parameters, (vi) prediction of secondary and tertiary structure, (vii) molecular docking to the immune receptors, and (viii) dynamics’ analysis of the complexes. Some studies also include further computational immune simulation to assess the vaccine’s ability to stimulate the immune response (Figure 4).

Each step of this protocol is quite elaborate and usually requires the usage of several tools/servers. As information about vaccine construction has not previously been addressed in computational reviews about TLRs, a brief summary is given here. Target sequences might be obtained from databases like PDB or UniProt.^71^ Then, they are submitted, e.g., to the VaxiJen^72^ to check the antigenicity and to ExpasyProtParam^73^ to investigate the physicochemical properties. Multiple servers can be used to predict the epitopes, depending on the type. Among them, there are NetCTL,^74^ NetMHCIIpan,^75^ Immune Epitope Database,^76^ BepiPred,^77^ and BCPREDS.^78^ Antigenicity, promiscuity, and allergenicity of epitopes can be evaluated with the use of AllerTop,^79^ AlgPred,^80,81^ VaxiJen, and ToxinPred^82,83^ servers. Structural evaluation of the vaccine begins with the prediction of secondary structure, which is usually done by the SOPMA server.^84^ Later, the tertiary structure can be predicted, often by the I-TASSER.^85^ However, the obtained models still need further refinement. For that, ModRefiner^86^ and GalaxyRefine^87^ are common choices. At this stage, it is evident that the way to obtain a structure of this type of modulator is quite demanding. Molecular docking of the epitope involves predicting the proper orientation and conformation of the epitope when it interacts with the immune receptor’s binding site. The ClusPro server^88^ is able to perform such computations. Further investigation of the dynamical properties is usually performed using Normal Mode Analysis (NMA) rather than all-atom MD simulations. However, the latter one (if used) can provide better and more detailed insight. A simulation of a possible immune response, which usually concludes the *in silico* part, is often performed using the C-ImmSim tool.^89^

In studying TLRs, molecular docking combined with the investigation of the dynamical stabilities and prediction of the vaccine’s ability to stimulate the immune response are the most crucial. The above-mentioned protocol and its variations have been used multiple times for vaccine design. Undoubtedly, vaccines against severe acute respiratory syndrome coronavirus 2 (SARS-CoV-2) have received the most attention in recent years.^90−93^ However, studies on other vaccine designs have also been carried out, both before and after the outbreak of COVID-19. The following examples are studies focused on designing vaccines against Middle East respiratory syndrome (MERS),^94^ Hepatitis C virus (HCV),^95^ human immunodeficiency virus (HIV),^96^ Neo-Coronavirus (NeoCoV),^97^ Human cytomegalovirus (HCMV),^98^ Kaposi Sarcoma,^99^ as well as infections like dengue,^100^ chikungunya,^101^ or those caused by *Taenia solium*,^102^*Klebsiella oxytoca*,^103^*Klebsiella pneumoniae*,^104^ or *Mycobacterium tuberculosis*.^105,106^ What is also worth mentioning in the context of next-generation vaccine design is the potential use of TLR agonists as vaccine adjuvants. Since TLR agonists are capable of stimulating innate immune responses, which also trigger adaptive immune responses, they can likewise be used to improve vaccine efficacy.^69,107,108^ For instance, monophosphoryl lipid A (MPL) and CpG-1018 have been used as adjuvants in licensed vaccines, and other TLR agonists are under the investigation.

Below, we want to elaborate more on vaccines against SARS-CoV-2, although the ultimate goal remains similar in all the studies—to get a stable protein-vaccine complex that triggers the immune response.

Different groups focused on studies of multiepitope vaccines against various TLRs. For instance, Oladipo et al.^90^ studied the TLR2, TLR3, TLR4, and TLR9, while Rafi et al.^91^ focused on TLR2 and TLR4, and Ysrafil et al.^92^ investigated TLR3, TLR4, and TLR8, as well as angiotensin-converting enzyme 2 (ACE2) as the entry receptors of SARS-CoV-2. Drawing upon the structure of the SARS-CoV-2 spike (S) glycoprotein (and nucleocapsid (N) protein and open reading frame 1a (ORF1a) protein in the case of Ysrafil et al.^92^), the authors tried to develop a potent multiepitope subunit vaccine. The groups received different predictions of the epitopes, depending on the particular settings used while executing the general protocol which was described earlier. Therefore, the final models of the multiepitope vaccine constructs were different, dependent on the sequences that build the individual epitopes. Here, we wanted to provide more details about the interesting study proposed by Pitaloka et al.^93^ The authors focused on designing a vaccine for protection against *Mycobacterium tuberculosis* (MTB) and SARS-CoV-2 coinfections. They used web servers—Bepipred-2.0 for B-cells epitopes, NetCTL.1.2 for Cytotoxic T Lymphocytes (CTL) epitopes, and Net MHC II pan 3.2 for Helper T Lymphocyte (HTL) epitopes—to screen potential epitopes from outer membrane protein A Rv0899 (OmpATb) of MTB and S protein of SARS-CoV-2. Epitope domains were selected from identified immunodominant areas and filtered out (by BLASTp) based on shared homology with humans. Then, at the vaccine’s N-terminus, the authors introduced the 50S ribosomal protein L7/L12 adjuvant using a commonly used EAAAK linker, while AAY and GPGPG linkers were used to connect the particular epitopes. In general, all the results showed that the proposed multiepitope vaccine candidates were nontoxic, capable of initiating the immunogenic response and not inducing an allergic reaction. Also, the molecular docking results revealed rather strong and stable interactions between the constructed vaccines and particular receptors within their LRR domains. During the computational simulations of the potential immune response using the C-ImmSim tool, the authors noticed a rise in the production of immune defenses, i.a. rise in the HTL cell population with memory T and B cells development, an increase in IgM, IgG1 + IgG2, and IgG + IgM antibody levels. The stability of the complexes of various vaccines was confirmed by studying their dynamic properties. For instance, Oladipo et al.^90^ and Pitaloka et al.^93^ used NMA to study the stability and mobility of selected receptor–vaccine complexes. In the first study, as a result, the vaccine protein and its receptor were predicted to spin toward each other. In the second study, based on the detected correlations in the covariance matrix between pairs of residues, the authors confirmed the stability of the vaccine candidate model. Rafi et al.^91^ performed classical MD simulations to check the stability of the constructed vaccine with the extracellular subunit of TLR2 and TLR4/MD2. The results indicated that the TLR–vaccine complexes were both stable and compact during the simulations. Especially for the TLR4–vaccine complex, a strong hydrogen bond network was pointed out, suggesting reduced flexibility of the vaccine when bound to the receptor, improved binding strength, and increased vaccine–receptor stability. Furthermore, the authors expanded their analysis by using the full-length heterodimer TLR4/MD2–vaccine complex, which was placed in a membrane to imitate the dynamic behavior during the MD simulation of the vaccine in biological systems. This study is one of the first where the full-length models of TLR receptors from the AlphaFold Protein Structure Database were used. For both TLR2 and TLR4 complexes, significant structural transitions toward membrane bilayer were observed, but the crucial interactions between the vaccine and the extracellular domain of receptors remained stable. Based on the observations made in the above-mentioned papers, one can speculate that during the binding, potentially well-designed vaccines may have a stabilizing effect on the TLRs in the system.

Although at first glance epitopes may be treated similarly to small-molecule modulators, the specificity of their search is quite different. It takes into account not only the process of binding to the TLR but also the stability and specificity of the epitope. Research on epitopes has the potential to reveal the mechanism of action of TLRs and their specificity to a greater extent. In the near future, this type of research can contribute to a much better understanding of the functioning of our immune system and the recognition of threats. We also anticipate that the contribution of AI-based methods will allow for a better understanding of the signaling pathways and their interrelations.

## Dynamic Nature of TLRs

The complexity of TLRs has consequences in the relatively weak understanding of the structural basis of their modes of action. Therefore, significant effort is required to comprehend TLR dynamics at the level of particular domains, the full-length receptor, and the dimerization process. Here, in the first part, we gave an outline of the studies that examined the effect of certain mutations on the receptor’s dynamics. In the second part, we summarized the works that focused on the characterization of the dynamical properties and conformational changes of full-length TLRs.

### Mutations’ Effects on the TLRs Dynamics

It is known that even a single mutation can induce substantial changes in terms of the macromolecule’s structure and function. For TLRs, one can hypothesize that depending on the mutation location, the ligand recognition or the adaptor protein binding could be disturbed. Below, we summarized studies focused on examining the effect of various mutations on TLRs. Those studies have usually focused on the analysis of individual domains of TLRs—the LRR or TIR domains.

Regarding the LRR domain, Anwar and Choi^109^ examined the structure–activity relationship in TLR4 mutants by the application of MD simulations (GROMACS with AMBER99SB-ILDN force field and TIP3P water model) together with principal component (PCA) and residue interaction network (RIN) analyses (RINalyzer, Cytoscape). To evaluate the influence of single nucleotide polymorphisms (SNPs), they examined four different models: (i) wild-type TLR4 (TLR4WT; PDB ID: 3FXI); (ii) a double mutant—aspartic acid-to-glycine at position 299 and threonine-to-isoleucine at position 399 (TLR4GI; PDB ID: 4G8A); (iii) the aspartic acid-to-glycine mutant (TLR4G299); and (iv) the threonine-to-isoleucine mutant (TLR4I399). Those mutations were classified as eliminating signaling activity; however, they did not disturb the ligand recognition nor did they establish contact with the associated MD2 protein. The single mutant structures were generated with the use of Chimera software. Computational studies revealed differences in the dynamic properties of the analyzed variants. The authors pointed out that the mutated complexes were less cohesive and displayed both local and global variation in the secondary structure, which could affect the proper exploration of conformational phase space. In particular, results from PCA confirmed that the mutated variants displayed unique low-frequency motions, which could be linked to the differential behaviors in these TLR4 variants. The authors also showed that decay in the rotational correlation function together with the observed density distributions and alteration of the number of hydrogen bonds between the protein and ligand could result in the loss of function.

Gosu et al.^110^ performed MD simulations (GROMACS with AMBER-ff99SB-ILDN force field and TIP3P water model) of human wild-type and mutant TLR3 to get insights into the dynamic nature of the dsRNA-bound TLR3 complex. They investigated several complexes: dsRNA-unbound TLR3 wild-type dimer (apo_dTLR3WT), dsRNA-bound TLR3 wild-type dimer (dTLR3WT-dsRNA), dsRNA-bound TLR3 dimer with a leucine-to-phenylalanine mutation at position 412 (dTLR3L412F-dsRNA), and dsRNA-bound TLR3 dimer with a proline-to-leucine mutation at position 680 (dTLR3P680L-dsRNA). In TLR3, L412F polymorphism was associated with several human diseases, while the P680L mutation was found as one that reduces the binding affinity of dsRNA to TLR3 and affects subsequent signaling. A human TLR3 dimer model was built by homology modeling using the mouse TLR3 dimer crystal structure (PDB ID: 3CIY) as a template to obtain an accurate structure conformation. The mutations were introduced using Discovery Studio Visualizer. The authors performed MD simulations (GROMACS with AMBER-ff99SB-ILDN force field and TIP3P water model) together with PCA, RIN, hydrogen bond, and protein-nucleic acid interaction analyses to investigate the global motions and the distribution of crucial residues for signal transduction. They claimed that the apo wild-type preformed dimer is unlikely to be stable in physiological conditions. Thus, they proposed that TLR3 might exist as a monomer in a solution. Further, the interaction energies and hydrogen bonds analyses indicated that the mutations induced certain conformational changes that could disturb the TLR3 signaling. The interaction sites between TLR3 and dsRNA were observed at both the N-terminal and C-terminal ends of TLR3 LRR, while the dimerization interface was confirmed at the C-terminal site but only for dTLR3WT-dsRNA and dTLR3L412F-dsRNA. It might suggest that P680 is crucial for maintaining the dimer interface for ligand binding. This hypothesis seems to be confirmed by the MD simulations in which the mutation dTLR3P608L disrupted the dimer interface in two out of three runs.

In the case of TLR3, we also want to underline one of the possible post-translational processes that the protein may undergo—glycosylation. TLR3 is a receptor with multiple glycosylation sites. Although most of these sites are not associated with dsRNA recognition, the *N*-glycan located at N413 has been observed to be in direct contact with viral dsRNA. In their work, Sun et al.^111^ reported that mutations of two independent glycosylation sites (N247and/or N413) in TLR3 resulted in the abolishing activity of ligand-induced TLR3 downstream signaling, which indicates that *N*-glycosylation at N413 is important in ligand recognition. Very recently, Wang et al.^112^ published a paper in which they analyzed the role of *N*-glycan in TLR3, specifically at the N413 position via both classical and umbrella sampling MD simulation (NAMD with CHARMM36m force field) combined with NMA. They prepared six systems to assess the stability of TLR3s: TLR3 (N413 unglycosylated) with/without dsRNA, TLR3 with the paucimannosidic glycan (N413-Man_3_GlcNAc_2_) with/without dsRNA, and TLR3 with the oligomannosidic glycan (N413-Man_9_GlcNAc_2_) with/without dsRNA. The authors used the glycosylated TLR3 LRR complexed with dsRNA from the PDB (PDB ID: 3CIY). For N413, glycosylation states were built using the Glycan Reader and Modeler module. The authors found that the loop region of LRR12 in TLR3 is important for interacting with dsRNA via the formation of hydrogen bonds. The glycan at N413 stabilized dsRNA in the TLR3 binding site and altered the dynamics of the binding process, with its size, length, and branch affecting the thermodynamics and dynamics of TLR3 recognition with dsRNA. These findings provide a new perspective for modulating TLR3 function and extend our understanding of the biological role of glycans in innate immune recognition.

Regarding the TIR domain, Mahita and Sowdhamini investigated the effect of key mutations on the conformational dynamics, based on TLR2 and TLR3.^113^ For that, they used a combination of MD simulations (GROMOS96 54a7 force field), protein–protein interaction (PPCheck), and protein structure network analyses. They carried out the analyses for eight different complexes, including not only wild-type and mutant dimers but also wild-type and mutant trimers (TIR dimers with different adaptor proteins). To build the complexes of the receptors with the adaptor proteins, the authors performed a protein–protein docking (HADDOCK). The following computational studies highlighted the significant differences between the dimer interfaces of the wild type and mutant forms and also provided a possible explanation of how the introduced mutations may affect adaptor binding to the receptor. For the proline-to-histidine (P681H) mutation in the TIR domain of TLR2, they observed an increase in the stability of the TLR1-TLR2 heterodimer. This mutation also affected the surface of the putative adaptor-binding platform causing it to become slightly more curved. For the alanine-to-proline (A795P) mutation in the TIR domain of TLR3, they pointed out that individual subunits in a mutant tilt slightly more toward each other in comparison to the wild type. Such a subtle change may influence the orientation of the BB-loops (important for mediating interactions between dimer subunits) on the homodimer, and thus also the binding of the adaptor proteins—MyD88 and TRIF. The authors pointed out that the obtained results were based on the assumption that TLR2 and the TLR3 TIR dimer adopt a similar conformation as that of the TLR10 TIR dimer crystal structure. As they admitted, this does not rule out the possibility of the dimers adopting a different TIR dimer conformation during signal transduction, e.g., an asymmetrical arrangement.

Ghosh et al.^114^ showed that by applying the random alanine scanning mutation (with Robetta, using Computational Interface Alanine Scanning Server), it was possible to validate how much the residues from the BB- and DD-loops of the TIR domain contribute to TLR2 heterodimer complex formation. For that, the binding free energy (ΔΔGbinding) of the interface residues was computed. The residues with positive cutoff values >0.5 kcal/mol were accepted as the residues of importance in the dimer stability for human TLR1–2 and TLR2–6. The authors concluded that for the hTLR1-TLR2 complex, three residues—Q97, N99, Y136 of TLR1— and two residues—E55, K62 of TLR2—impact the binding energy of the complex. For the hTLR2-TLR6 complex, the following residues were predicted to have a significant role: Y44, W45 of TLR2 and E159, K160 of TLR6. While combining the results of alanine scanning mutation studies with sequence alignment, structure prediction and superimposition, molecular docking (ZDOCK), and MD simulations (GROMACS with GROMOS96 54a7 force field and SPC water model), the authors presented two key conclusions. The first was that the subtle conformational variations in the TLR structures might play a crucial role during special circumstances. The second was that the role of TLR2 BB-loop residues and TLR1/TLR6 near-DD-loop residues is important for the process of heterodimerization and for initiating differential downstream signaling.

In the summarized studies,^109,110,113,114^ the authors showed that the analysis of mutations’ effect can be helpful not only in studying the TLRs’ structural dynamics but also in uncovering their mechanism of action, especially in the context of ligand or adaptor protein binding. However, we still have limited knowledge regarding the particular TLRs. Given the fact that many more mutations in TLRs are reported (e.g., in the UniProt or ClinVar^115^ databases), more research should be carried out to clarify the effect of those substitutions.

### Full-Length TLRs

**Figure 5:**
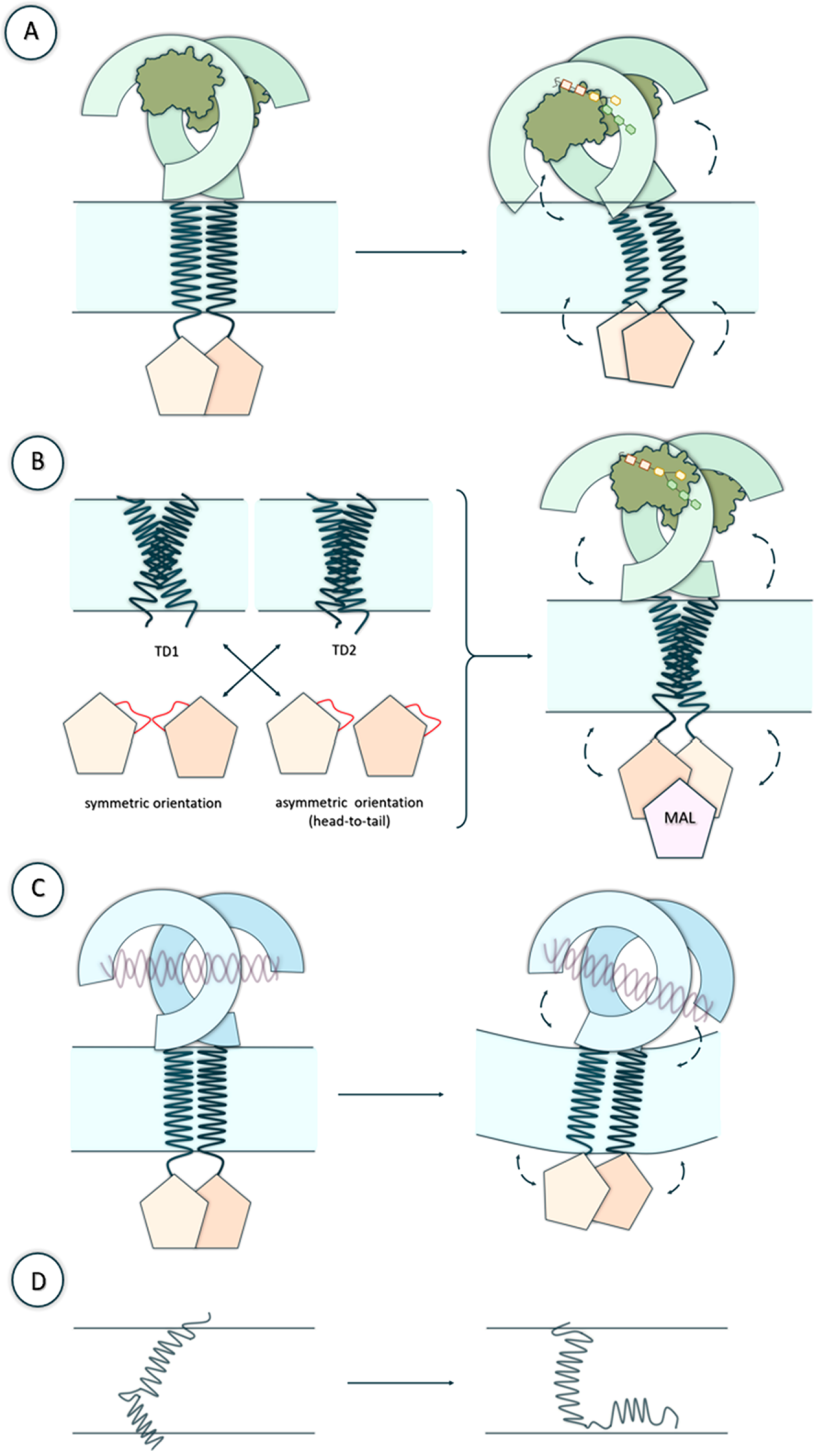
Examples of potential dynamical changes of TLRs observed in cited studies. (A and B) Structural transitions that the particular domains of TLR4 may exhibit (based on Patra et al.^116^ and Matamoros-Recio et al.^117^ works). TLR4 was shown with the associated myeloid differentiation factor 2 (MD2) and with the bound lipopolysaccharide LPS (C) Structural rearrangements of TLR3 domains and membrane (based on Patra et al.^118^ study). TLR3 was shown bound with dsRNA. (D) Differences in the structural organization of the transmembrane helix (TM) and cytoplasmic juxtamembrane (JM) regions that may occur in TLRs (based on Kornilov et al.^34^ work).

Due to the complexity of the TLR structure and the presence of the lipid bilayer, the study of the dynamics of the full-length receptor is difficult. However, some studies have been published in recent years and they provided important insights, especially regarding the possible structure rearrangement and mechanism of action of TLRs. In Figure 5 we present the dynamical changes that particular TLRs can undergo which have been revealed and described in the recent years.

One of the first extensive studies of a full-length TLR in a membrane-aqueous environment was the work by Patra et al.^116^ The authors focused on TLR4 (TLR4/MD2/LPS homoheterodimer; TLR4 associated with MD2 protein and lipopolysaccharide LPS) and provided key insights into the orientation and interaction of LRR (named ECD in the paper), TM, and TIR domains with respect to the dipalmitoylphosphatidylcholine (DPPC) bilayer. To reach these results, they successfully applied homology modeling methods, followed by protein–protein docking and MD simulations. Additionally, they used molecular docking and binding free energy calculations to get insight into the binding of the TAK-242 ligand with the TLR4-TIR dimer. For each of the domains, the protocol had to be adapted accordingly to obtain the best possible models that could be included in a full-length structure. For instance, the dimeric LPS-bound LRR structure was obtained from the PDB (PDB ID: 3FXI), and missing residues were added (SWISS-MODEL), while the TM domain was modeled as a single α-helix and protein–protein docking (ZDOCK) was carried out to obtain a dimeric structure. The TIR domain was obtained via homology modeling using the crystal structure of TLR10 (PDB ID: 2J67) and consecutive superimposition of monomeric TLR4-TIR over the two subunits of dimeric TLR10-TIR resulted in a dimeric TLR4-TIR domain. Then, all three individual domains were aligned on a straight axis and peptide bonds were patched between the extreme C- and N-terminal residues to adjacent domains (Discovery Studio). The constructed model could be finally inserted into the pre-equilibrated bilayer and used for further MD simulation (GROMACS with Gromos96 54a7 and Barger-lipid hybrid force field and SPC water model) and molecular docking. The authors showed that each domain of TLR4 exhibits several structural transitions (Figure 5A). The results revealed that LRR and TIR domains may be partially immersed in the membrane bilayer and that the TM domain tilts and bends to overcome the hydrophobic mismatch with the bilayer core. The authors claimed that the dynamic properties of TLR4-LRR had little effect on the interactions between LPS and MD2. For the TLR4-TM, the authors pointed out the possibility of an alternate dimerization or a potential oligomerization interface, as previously found for TLR3-TM.^30^ Patra et al. also observed that the gradual absorption of the TLR4-TIR domain to the membrane leaflet could be a consequence of the electrostatic interactions and the bending/twisting actions of the LRR and TM domains. Their analyses indicated that even though TLR4-TIR surfaces are potentially membrane-absorbed, they also include the solvent-exposed part dedicated to interactions with other proteins. Thus, such a partial immersion is unlikely to prevent these segments from contacting the adaptor or other binding components. In the case of TLR4, the MyD88 adaptor protein is guided to TLR4-TIR by the membrane-anchored adaptor, TIR domain-containing adaptor protein (TIRAP). Hence, it is probable that the activated receptor complex TLR4/TIRAP/MyD88 is close to the membrane. For TAK-242, Patra et al. constructed two possible homodimerization interfaces—first, where helix αC and the BB loop of both TIR subunits form the dimer interface, and second, where helix αC is exposed toward the solvent and places helix αE and the BB loop in between the dimer interface. Results obtained from estimated binding free energy revealed that the first model—the αC-αC dimer—had a greater binding affinity and that the affinity of TAK-242 for the αC-αC dimer was stronger than for the αE-BB dimer. This could be an indication that the αC-αC/BB-BB model might represent the physiological dimeric interface of TLR4. However, the TAK-242 binding inside the TIR dimer cavity remains speculative, since in the case of separate simulation of full-length TLR4 as well as simulation of full-length TLR4 with TAK-242, the binding cavity of the ligand was partially blocked due to the rotation and upward movement of the TIR dimer.

In the following years, Matamoros-Recio et al.^117^ also studied the full-length model of the agonist LPS-bound TLR4. The complete model was obtained from combining the individual domains that were previously optimized by different protocols. For the dimeric LPS-bound LRR structure, the crystal structure (PDB ID: 3FXI) was selected and optimized (Maestro), while the structure of the TM domain was predicted by submitting their sequences to TMDOCK and PREDDIMER web servers. Finally, the homology modeling (implemented in YASARA) was used to predict the TIR domain dimer, using the tempates of the TIR domains of human TLR1, TLR2, TLR6, and TLR10. The authors combined *ab initio* calculations with molecular docking, all-atom MD simulations, and thermodynamics calculations to provide the complete 3D models of the active TLR4 complex embedded into a membrane system. In total, they analyzed four full-length models, different dimerization interfaces for TM domain and orientations of TIR domain were observed (Figure 5B). They showed that the interactions on different interfaces—TLR4/TLR4*, TLR4/MD-2*, and TLR4*/MD2—were kept within the simulations and that both subunits in the dimeric complex show a mutual stabilizing role. Also, they confirmed that the transmembrane domain and the following hydrophobic region (HR) indicate plasticity, depending on the membrane composition. Such plasticity may determine the dimerization of the intracellular domain. These observations are supported by a recent study by Kornilov et al.^34^ in which the results of MD simulations (GROMACS with AMBER ff14SB and slipids force fields and TIP3P water model) indicated that juxtamembrane (JM) regions of various TLRs interact with lipids and are immersed into the bilayer membrane. The simulations showed that both TM and JM generally retain their secondary structure but adapt to the nonpolar environment by changing their tilt to the membrane and by rotating to find the optimal location of charged and nonpolar residues at the lipid–water interface (Figure 5D). In their study, Matamoros-Recio et al.^117^ proposed two models of TM-TM* (named TD-TD* in the paper) and pointed out that TM-HR can adopt different conformations, thus changing the mode of dimerization depending on the environment, regulated by TLR4 localization. The authors described also two models for the TIR-TIR* dimer (named ID-ID* in the paper)—symmetrical and asymmetrical. In the first model, the αC helix and the BB-loop in TIR domains were facing the dimerization interface, while in the second model, the dimerization interface was preserved in a head-to-tail way. The authors pointed out that both models were capable of binding the adaptor proteins. It could mean that the dimerization mechanism, and thus the receptor’s activation depends on (among others) the membrane composition (localization of TLR4) and structural rearrangement. They also showed that both symmetric and asymmetric TIR-TIR* models are suitable for MyD88-adapter-like (MAL) binding, supporting the hypothesis that both models could coexist, and have a direct implication in the activation of distinct TLR4 pathways.

In their other work, Patra et al. studied the structure and dynamics of a full-length dimer of TLR3 immersed in a bilayer of 1-palmitoyl-2-oleoyl-*sn*-glycero-3-phosphocholine (POPC).^118^ They used a similar set of molecular modeling methods as in the case of TLR4.^116^ They studied three membrane-solvated complexes of the TLR3 homodimer bound with the dsRNA. Their analyses indicated that the TLR3-TIR homodimer built from the TLR6-TIR structure led to obtaining a full-length receptor structure with the stability necessary to maintain key intermolecular interactions with the ligand and with the membrane. Furthermore, they showed that flexible juxtamembrane loops of TLR3 allow for the simultaneous bending of the LRR and TIR domains on both surfaces of the membrane. They also observed that the complex immersed in the bilayer progressively tilted on the bilayer surface due to the electrostatic attraction between the charged parts of both the protein and phospholipids from the bilayer (Figure 5C). In that case, the LRR-NT was only partially absorbed by the lipid headgroups. That was in contrast to the LRR-NT from their previously reported TLR4 model that was completely buried in the bilayer surface. They assumed that it is possible that the negatively charged dsRNA restricted the insertion of LRR-NT into the membrane surface. During the simulations, the dsRNA kept its structural integrity while bound to TLR3. The observed distortions in the TLR3-TM domain were distinct from the previously reported TLR4-TM. Thus, the authors concluded that the orientation and conformational changes of each TLR type may vary, depending on their location in the cell or the lipid composition in the membrane. Based on the MD simulations analysis, Patra et al. indicated the probable interface involving residues from the αC and αD helix and the CD and DE loops of both TIR monomers. The BB-loop of one subunit was completely solvent-exposed, while the other was partially involved in dimer packing. The solvent-exposed part confirmed the importance of this segment in TRIF recruitment by the activated receptor.

The reviewed papers revealed important insight into TLRs dynamics. In summarized studies, the authors presented relevant information on possible changes in position and conformation that receptors embedded in the cell membrane or intracellular compartments may undergo. Also, an important message regarding the potential mechanism of TIR domain dimerization and binding of the adaptor protein came from the analyzed models of both symmetrical and asymmetrical domains. This may be helpful for designing new types of TLR modulators, especially those targeting the TIR domain. One should remember that the presented studies on full-length receptors refer only to TLR3 and TLR4, which means that for now, the conclusions cannot be unified for all other receptors. As in the case of studying the effect of mutations, it seems that research regarding the dynamics of TLRs is just beginning. Considering the differences in TLRs structure, substrate recognition, dimerization requirements, and association with adaptor proteins, along with the importance of understanding the TLRs signal transduction pathway, we can expect a significant increase in interest in this field in the coming years.

## Conclusions

Toll-like receptors are one of the most crucial components of the immune system. Given their importance, it was not a surprise that the 2011 Nobel Prize in Physiology or Medicine was awarded to Dr. Jules A. Hoffmann and Dr. Bruce A. Beutler for their discoveries of the role of TLRs in innate immunity. It happened relatively quickly after the discovery of TLRs, only within 15 years. Since that time, tens of thousands of papers have been published in which TLRs have been the main subject of research. TLRs are complicated in terms of their structure, dynamics, and functioning, and this complexity is a challenge despite the enormous progress in the development of both experimental and computational methods. In our review, we aimed to highlight the progress made in recent years with the use of *in silico* methods for TLRs studies. Also, we wanted to point out the areas that still await their discoverers. One of the main limitations in understanding the function of TLRs is difficulty in the proper characterization of receptor structure at various stages of signal transduction. Even the latest breakthrough in AI-based structure prediction is not yet widely used in research aimed at revealing the mechanism of action of TLRs.

Based on the results presented in the reviewed papers, we can conclude that still, the most attention is paid to the use of computational solutions for the design of small-molecule modulators. The use of *in silico* methods to design other types of modulators, such as multiepitope vaccines, is gaining more popularity, but yet, it is not as common as in the case of small-molecule compounds. Both small-molecule and multiepitope modulators are designed in such a way as to target the LRR of TLRs. There was no breakthrough in the design of small-molecule modulators targeting the TIR domain. Among things that scientists will want to keep improving is obtaining the best binding affinity and stability of the modulators. Regarding the dynamics of TLRs, scientists have shown that studying the mutations’ effect can contribute to a better understanding of the potential mechanism of action of the receptors. That is of special interest for both ligand and adaptor protein binding. More demanding, both in terms of system preparation and computing power, is the analysis of the dynamics of the full-length TLR complex. So far, only TLR3 and 4 have been built as full-length models embedded in the lipid bilayer. Those studies presented relevant information on possible conformational changes that may occur in the receptor’s structure. Thus, it would be very important to perform similar studies for members of the TLR family. Since now we have easier access to the predictions of large macromolecule structures, we expect that in the coming years, we will witness progress in research on the TLRs’ dynamics and mechanism of action.

**Figure 6:**
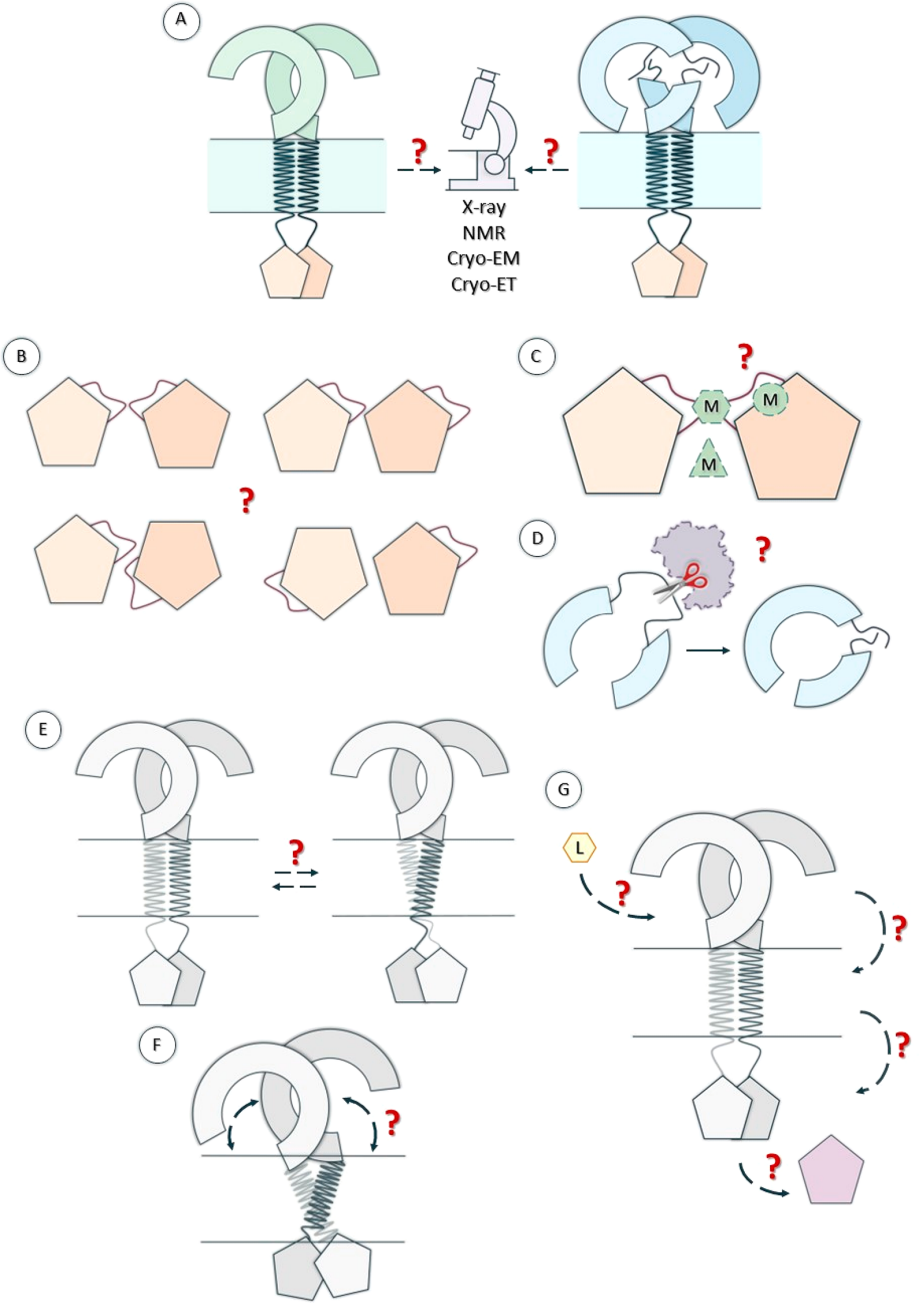
Areas in TLR research that still require further development. (A) Experimental verification of the predicted structures. (B) Studying the orientation of the subunits of the TIR domain dimers of TLRs. (C) Designing small-molecule modulators (M) targeting the TIR domain of TLRs. (D) Studying the proteolytic cleavage of the Z-loop in TLR7–9. (E) Analyzing potential changes in the subunits dynamics in TLRs. (F) Analyzing the conformational changes and structural rearrangements in both TLR receptors and bilayer membrane. (G) Studying the whole process of ligand recognition through the signaling cascade to the immune response.

In Figure 6 we presented the main areas in TLR research that still require further studies. Figure 6A illustrates the necessity of the experimental verification of the predicted structures. Despite the great progress in AI-based methods to predict the tertiary structures of macromolecules, experimental validation is a must to confirm the compliance of the obtained predictions. Access to experimentally solved structures of transmembrane proteins is also important in order to confirm the orientation of individual domains or subunits of the structure toward each other. Obtaining information about the orientation of the subunits of the TIR domain dimers of TLRs is of special interest (Figure 6B). So far, we have information about possible symmetrical or asymmetric orientations. However, we lack a systematic review of what orientations are preferred by specific receptors and how the orientation of the subunits can determine the binding of the adaptor proteins and the initiation of the signal cascade. This issue is also related to the design of small-molecule modulators targeting the TIR domain (Figure 6C). Without details about the orientation of the subunits, it is difficult to properly select the best binding site for modulators.

As we mentioned in the Introduction of this review, some TLRs (7–9) require the proteolytic cleavage of the Z-loop in their LRR domain (Figure 6D). This is needed to allow ligands to bind and to further activate the receptor. Very little is known about the molecular basis of this process. Basically, only the information about the examples of proteases potentially involved in cleavage is available. To our best knowledge, there are no *in silico* studies attempting to explain this process. We are aware that one of the obstacles may be the size of the system and that no accurate structure predictions of the TLR-protease complex have been available so far. However, we hope that with the increase of the computational resources and the possibility to predict the structure of complexes using, e.g., AlphaFold Multimer, this issue will be soon addressed.

In Figure 6E,F, we wanted to highlight the importance of conducting further research on the dynamics and conformational changes of TLRs. As we mentioned, studies presented to date have mainly focused on TLR3 and TLR4. Very little is known about other receptors, e.g., how the conformational changes occur in individual subunits or how full-length receptors behave in relation to the membrane in which they are immersed. In particular, we would like to know whether the location of the receptor (cell membrane or intracellular compartments) determines the TLRs’ dynamics and the subsequent ability to bind the adaptor proteins. Figure 6G illustrates the ultimate goal of studying the Toll-like receptors with the use of computational methods, which is to get deep insight into each stage of the receptor functioning. Thus, the challenge is to combine all the information, starting from the recognition of the ligand by the receptor, through the triggering of the signaling cascade, to the immune response.

## Data Availability

Information about human Toll-like receptors domains deposited in the Protein Data Bank and information about chemical structures of the best hits together (small-molecule agonists and antagonists) with the identified chemical interactions from the reviewed research papers are provided in the Supporting Information.

## Supporting Information Available

Supplementary Table S1. Overview of human Toll-like receptors domains deposited in the Protein Data Bank. Supplementary Table S2. Chemical structures of the best hits (small-molecule agonists and antagonists) from the reviewed research papers. (PDF)

## Abbreviations

TLRs: Toll-like receptors
PRRs: pattern recognition receptors
MPs: molecular patterns
DAMPs: damage/danger-associated molecular patterns
MAMPs: microbial/microbe-associated molecular pattaerns
PAMPs: pathogen-associated molecular patterns
XAMPs: xenobiotic-associated molecular patterns
LRR: leucine-rich repeats domain
TM: transmembrane domain
TIR: Toll-interleukin-1 receptor domain
MyD88: myeloid differentiation primary-response protein 88
TRIF: TIR domain-containing adaptor protein inducing interferon-β
hTLRs: human Toll-like receptors
PDB: Protein Data Bank
JM: juxtamembrane
VS: virtual screening
MD: molecular dynamics
MM-PBSA: Molecular Mechanics Poisson–Boltzmann Surface Area
MM-GBSA: Molecular Mechanics with Generalized Born and Surface Area
NF-κB: nuclear factor kappa-light-chain-enhancer of activated B cells
SAR: structure–activity relationship
MD2: myeloid differentiation factor 2
ADMET: absorption, distribution, metabolism, excretion, toxicity properties
IL: interleukine
METH: methamphetamine
NMA: Normal Mode Analysis
SARS-CoV-2: severe acute respiratory syndrome coronavirus 2
MERS: Middle East respiratory syndrome
HCV: Hepatitis C virus
HIV: human immunodeficiency virus
NeoCoV: Neo-Coronavirus
MPL: monophosphoryl lipid A
S protein: SARS-CoV-2 spike glycoprotein
N protein: nucleocapsid protein
ORF1a: open reading frame 1a protein
MTB: *Mycobacterium tuberculosis*
OmpATb: outer membrane protein A Rv0899
HTL: human thymus lymphoid
IgM: immunoglobulin M
IgG: immunoglobulin G
PCA: principal component analysis
RIN: residue interaction network
SNPs: single nucleotide polymorphisms
WT: wild type
LPS: lipopolysaccharide
DPPC: dipalmitoylphosphatidylcholine bilayer
POPC: 1-palmitoyl-2-oleoyl-*sn*-glycero-3-phosphocholine
TIRAP: TIR domain-containing adaptor protein
