# Supplementary material for: Recent Advances in Studying Toll-like Receptors with the Use of Computational Methods
Source: J Chem Inf Model. 2023 Jun 7;63(12):3669–87. doi: 10.1021/acs.jcim.3c00419 (PMC10302489; doi:10.1021/acs.jcim.3c00419)
Supplement: Supplementary file 1 — ci3c00419_si_001.pdf [file ci3c00419_si_001.pdf]

Supporting Information

Recent advances in studying Toll-like receptors with the use of computational methods

Maria Bzówka[1,2], Weronika Bagrowska[1], Artur Góra[1]*

[1]Tunneling Group, Biotechnology Centre, Silesian University of Technology, Gliwice, Poland

[2] Department of Organic Chemistry, Bioorganic Chemistry and Biotechnology, Faculty of Chemistry, Silesian University of Technology, Gliwice, Poland

Corresponding Author
*Maria Bzówka maria.bzowka@polsl.pl or m.bzowka@tunnelinggroup.pl

Supplementary Table S1. Overview of human Toll-like receptors domains deposited in the Protein Data Bank.

TLR member	PDB ID	Year	Resolution [Å] (method)	Domain(s)	Mutation(s)	Other macromolecules, small molecules and biologically interesting molecules	Oligo-saccharides
TLR1	6nih	2019	2.30 (X-ray)	LRR *hybrid of human TLR1 and hagfish variable lymphocyte receptor B (VLRB)	No	2-acetamido-2-deoxy-beta-D-glucopyranose (NAG), triacetyl-beta-chitotriose (PRD_900017)	Yes
	2z7x	2007	2.10 (X-ray)	LRR *hybrid of human TLR1 and hagfish VLRB	No	synthetic triacylated lipopeptide (Pam3CSK4), (2R)-3-{[(2S)-3-hydroxy-2-(palmitoylamino)propyl]thio}propane-1,2-diyl dihexadecanoate (PCJ), NAG	Yes
	7nt7	2021	Solution NMR	TIR	No	-	No
	7nuw	2021	1.90 (X-ray)	TIR	No	-	No
	7nux	2021	2.47 (X-ray)	TIR	No	-	No
	1fyv	2000	2.90 (X-ray)	TIR	Yes	selenomethionine (MSE)	No
TLR2	6nig	2019	2.35 (X-ray)	LRR *hybrid of human TLR2 and hagfish VLRB	No	(3S,4S,3'S,4'S)-1,1'-(1,4-phenylenedicarbonyl)bis{N~3~,N~4~-bis[(1S,2R)-2-phenylcyclopropyl]pyrrolidine-3,4-dicarboxami de} (KQD), NAG	Yes
	2z80	2007	1.80 (X-ray)	LRR *hybrid of human TLR2 and hagfish VLRB	No	NAG	No
	2z7x	2007	2.10 (X-ray)	LRR *hybrid of human TLR2 and hagfish VLRB	No	Pam3CSK4, PCJ, NAG	Yes

	8ar0	2023	Solution NMR	TM + cytoplasmic juxtamembrane regions	No	-	No
	1o77	2002	3.20 (X-ray)	TIR	Yes, C713S	-	No
	1fyw	2000	3.00 (X-ray)	TIR	Yes	S-(dimethylarsenic)cysteine (CAS), MSE	No
	1fyx	2000	2.80 (X-ray)	TIR	Yes, P681H	CAS	No
TLR3	7wv3	2022	2.26 (Elencton microscopy)	LRR (TLR3 linear cluster)	No	synthetic construct dsRNA, NAG	Yes
	7wv4	2022	3.35 (Elencton microscopy)	LRR (ectoTLR3-poly(I:C) cluster)	No	synthetic construct dsRNA	No
	7wv5	2022	3.10 (Elencton microscopy)	LRR (ectoTLR3-poly(I:C)	No	synthetic construct dsRNA, NAG	Yes
	7wve	2022	3.11 (Elencton microscopy)	LRR (TLR3-poly(I:C) complex)	Yes, D523K,D524K,E527K	synthetic construct dsRNA	No
	7wvf	2022	3.91 (Elencton microscopy)	LRR (ectoTLR3-mAb12-poly(I:C) complex)	No	synthetic construct dsRNA, mAb12	No
	7wvj	2022	3.26 (Elencton microscopy)	LRR (TLR3 -poly I:C complex)	Yes, K117D,K139D,K145D	sythetic construct dsRNA	No
	5gs0	2016	3.27 (X-ray)	LRR	No	light chain (anti-TLR3), heavy chain (anti-TLR3), heavy chain (anti-Lid), light chain (anti-Lid); NAG, alpha-D-mannopyranose (MAN), beta-D-mannopyranose (BMA)	No
	3ulu	2012	3.52 (X-ray)	LRR	No	Fab15 light chain, Fab15 heavy chain, Fab12 light chain, Fab12 heavy chain, Fab1068 light chain, Fab1068 heavy chain, NAG, SO4	Yes
	3ulv	2012	3.52 (X-ray)	LRR	No	Fab15 light chain, Fab15 heavy chain, Fab12 light chain, Fab12 heavy chain, Fab1068 light chain, Fab1068 heavy chain, NAG, SO4	Yes

	1ziw	2005	2.10 (X-ray)	LRR	No	NAG, SO4, glycerol (GOL)	Yes
	2a0z	2005	2.40 (X-ray)	LRR	No	NAG, alpha-D-glucopyranose (GLC), SO4, beta-mercaptoethanol (BME)	Yes
	7c76	2021	3.40 (Cryo-EM)	LRR, TM	No	protein unc-93 homolog B1 (UNC93B1), NAG	Yes
	8ar1	2023	Solution NMR	TM + cytoplasmic juxtamembrane regions	No	-	No
	2mk9	2014	Solution NMR	TM	No	-	No
	2mka	2014	Solution NMR	TM	No	-	No
TLR4	3ul7	2012	2.37 (X-ray)	LRR *hybrid of human TLR4 and hagfish VLRB.61	Yes, F63W	NAG, beta-L-fucopyranose (FUL), SO4	Yes
	3ul8	2012	2.50 (X-ray)	LRR *hybrid of human TLR4 and hagfish VLRB.61	Yes, V134L	NAG, FUL, SO4	Yes
	3ul9	2012	2.45 (X-ray)	LRR *hybrid of human TLR4 and hagfish VLRB.61	Yes, M41E	NAG, FUL, SO4	Yes
	3ula	2012	3.60 (X-ray)	LRR *hybrid of human TLR4 and hagfish VLRB.61	Yes, F63W	lymphocyte antigen 96, 3-O-decyl-2-deoxy-6-O-{2-deoxy-3-O-[(3R)-3-methoxydecyl]-6-O-methyl-2-[(11Z)-octadec-11-enoylamino]-4-O-phosphono-neta-D-glucopyranosyl}-2-[(3-oxotetradecanoyl)amino]-1-O-phosphano-alpha-D-glucopyranose (E55), NAG,	Yes
	4g8a	2012	2.40 (X-ray)	LRR	Yes, D299G, T399I	lymphocyte antigen 96, 2-deoxy-3-O-[(3R)-3-hydroxytetradecanoyl]-2-{[(3R)-3-hydroxytetradecanoyl]amino}-4-O-phosphono-beta-D-glucopyranose (LP4), (R)-((2R,3S,4R,5R,6R)-3-hydroxy-2-(hydroxymethyl)-5-((R)-3-hydroxytetradecanamido)-6-(phosphonooxy)tetrahydro-2H-pyran-4-yl) 3-hydroxytetradecanoate (LP5), 3-deoxy-alpha-D-manno-oct-2-ulopyranosonic acid (KDO), myristic acid	Yes

						(MYR), laurin acid (DAO), NAG	
	3fxi	2009	3.10 (X-ray)	LRR	No	lymphocyte antigen 96, 3-hydroxy-tetradecanoic acid (FTT), MYR, NAG, DAO, PO4, MG	Yes
	2z62	2007	1.70 (X-ray)	LRR *hybrid of human TLR4 and hagfish VLRB.61	No	-	Yes
	2z63	2007	2.00 (X-ray)	LRR *hybrid of human TLR4 and hagfish VLRB.61	No	-	Yes
	2z65	2007	2.70 (X-ray)	LRR *hybrid of human TLR4 and hagfish VLRB.61	No	lymphocyte antigen 96, E55, NAG	Yes
	2z66	2007	1.90 (X-ray)	LRR *hybrid of human TLR4 and hagfish VLRB.61	No	SO4	Yes
	5nao	2018	Solution NMR	TM	No	-	No
	5nam	2017	Solution NMR	TM	No	-	No
TLR5	8ar2	2023	Solution NMR	TM + cytoplasmic juxtamembrane regions	No	-	No
	3j0a	2011	26.0 (Electron microscopy)	LRR, TM, TIR	No	-	Yes
TLR6	4om7	2014	2.20 (X-ray)	TIR	No	-	
TLR7	7cyn	2021	4.20 (Electron microscopy)	LRR, TM	No	protein unc-93 homolog B1 (UNC93B1)	Yes
TLR8	7rc9	2022	2.76 (X-ray)	LRR	No	2-(2,6-dimethylpyridin-4-yl)-5-(piperidin-4-yl)-3-(propan-2-yl)-1H-indole (RQY), NAG	Yes

	7r52	2022	2.94 Å(X-ray)	LRR	No	5-methoxy-6-pyridin-4-yl-1~{H}-indole, NAG	Yes
	7r53	2022	3.12 (X-ray)	LRR	No	5-cyclopropyl-6-(2,6-dimethylpyridin-4-yl)-~{N}-[(3~{R},4~{R})-3-fluoranylpiperidin-4-yl]-1~{H}-indazol-3-amine (I5N), NAG	Yes
	7r54	2022	2.84 (X-ray)	LRR	No	(5-methoxy-6-pyridin-4-yl-1~{H}-indazol-3-yl)-(4-methylpiperazin-1-yl)methanone (I5B), NAG	Yes
	6wml	2021	2.50 (X-ray)	LRR	No	(2R)-2-[(2-amino-7-fluoropyrido[3,2-d]pyrimidin-4-yl)amino]-2-methylhexan-1-ol (U57, GS-9688, selgantolimod), NAG, MAN	Yes
	7crf	2021	2.89 (X-ray)	LRR	No	1-[2-(ethoxymethyl)-4-iodanyl-5-phenyl-imidazol-1-yl]-2-methyl-propan-2-ol (GD0, CU-CPD107), NAG	Yes
	6kya	2020	2.89 (X-ray)	LRR	No	3-[5-methylsulfanyl-4-(3,4,5-trimethylphenyl)-1,2,4-triazol-3-yl]propan-1-ol (DY3, TH1027), NAG	Yes
	6ty5	2020	2.79 (X-ray)	LRR	No	5-methyl-7-(7-methyl-2-piperidin-4-yl-indazol-5-yl)furo[3,2-c]pyridin-4-one (O0W), NAG	Yes
	6v9u	2020	2.65 (X-ray)	LRR	No	2-(3,4-dimethoxyphenyl)-5-(piperidin-4-yl)-3-(propan-2-yl)-1H-indole (QSM), NAG	Yes
	6zjz	2020	2.49 (X-ray)	LRR	No	5-[(3~{R},5~{S})-3-azanyl-5-(trifluoromethyl)piperidin-1-yl]quinoline-8-carbonitrile (QLH), formic acid (FMT), NAG	Yes
	5z14	2018	2.80 (X-ray)	LRR	No	4-(7-methoxyquinolin-4-yl)-2-methyl-phenol (7VC), NAG	Yes
	5z15	2018	2.90 (X-ray)	LRR	No	4-(7-chloranylquinolin-4-yl)-2-methyl-phenol (7VL), NAG, MAN	Yes
	5wyx	2017	2.40 (X-ray)	LRR	No	7-(3-methylphenyl)pyrazolo[1,5-a]pyrimidine-3-carboxamide (CU8), NAG	Yes
	5wyz	2017	2.30 (X-ray)	LRR	No	4-(3-methyl-4-oxidanyl-phenyl)quinolin-7-ol (7VF), NAG	Yes
	4r6a	2016	2.10 (X-ray)	LRR	No	1-(4-amino-2-butyl-1H-imidazo[4,5-c]quinolin-1-yl)-2-methylpropan-2-ol (HB2), NAG	Yes
	5awa	2016	2.20 (X-ray)	LRR	No	5-[[3-(aminomethyl)phenyl]methyl]-3-pentyl-quinolin-2-amine (M8D), NAG	Yes

	5awc	2016	2.50 (X-ray)	LRR	No	5-(4-azanylbutyl)-3-pentyl-quinolin-2-amine (M4D), NAG	Yes
	5az5	2016	2.40 (X-ray)	LRR	No	1-pentyl-4-phenyl-imidazol-2-amine (MBL), NAG	Yes
	5hdh	2016	2.60 (X-ray)	LRR	Yes, R452N, K453Q, R454S, R455N	NAG, 2-(N-morpholino)-ethanesulfonic acid (MES), SO4	Yes
	4r07	2015	2.00 (X-ray)	LRR	No	3'-O-[(R)-{[(2R,3aR,4R,6R,6aR)-6-(2-amino-6-oxo-1,6-dihydro-9H-purin-9-yl)-2-hydroxy-2-oxidotetrahydrofuro[3,4-d][1,3,2]dioxaphosphol-4-yl]methoxy}(hydroxy)phosphoryl]uridine 5'-(dihydrogen phosphate) (UCG), uridine (URI), NAG	Yes
	4r08	2015	2.40 (X-ray)	LRR	No	3'-O-[(R)-{[(2R,3aR,4R,6R,6aR)-6-(2-amino-6-oxo-1,6-dihydro-9H-purin-9-yl)-2-hydroxy-2-oxidotetrahydrofuro[3,4-d][1,3,2]dioxaphosphol-4-yl]methoxy}(hydroxy)phosphoryl]uridine 5'-(dihydrogen phosphate) (UCG), uridine (URI), NAG	Yes
	4r09	2015	2.62 (X-ray)	LRR	No	O-[(2R,3S,4R,5R)-5-(2-amino-6-oxo-3,6-dihydro-9H-purin-9-yl)-2-({[(S)-({(2R,3S,4R,5R)-5-(2,4-dioxo-3,4-dihydropyrimidin-1(2H)-yl)-4-hydroxy-2-[(thiophosphonooxy)methyl]tetrahydrofuran-3-yl}oxy)(sulfanyl)phosphoryl]oxy}methyl)-4-hydroxytetrahydrofuran-3-yl] dihydrogen (S)-phosphorothioate (06S), 1-[(2R,3aR,4R,6R,6aR)-2-hydroxy-6-(hydroxymethyl)-2-sulfidotetrahydrofuro[3,4-d][1,3,2]dioxaphosphol-4-yl]pyrimidine-2,4(1H,3H)-dione (UPT), URI, NAG	Yes
	4r0a	2015	1.90 (X-ray)	LRR	No	URI, NAG	Yes
	5awb	2015	2.10 (X-ray)	LRR	No	1-[[3-(aminomethyl)phenyl]methyl]-2-butyl-imidazo[4,5-c]quinolin-4-amine (M0A), NAG	Yes
	5awd	2015	2.05 (X-ray)	LRR	No	1-[[4-(aminomethyl)phenyl]methyl]-2-butyl-imidazo[4,5-c]quinolin-4-amine (IDQ), NAG	Yes
	3wn4	2014	1.81 (X-ray)	LRR	No	2-butylfuro[2,3-c]quinolin-4-amine (D87), NAG	Yes
	4qbz	2014	2.00 (X-ray)	LRR	No	2-butyl[1,3]oxazolo[4,5-c]quinolin-4-amine (D80), NAG	Yes
	4qc0	2014	2.10 (X-ray)	LRR	No	2-butyl-2H-pyrazolo[3,4-c]quinolin-4-amine (XG1), NAG	Yes

	3w3g	2013	2.30 (X-ray)	LRR	No	(R,R)-2,3-butanediol (BU3), NAG	Yes
	3w3j	2013	2.00 (X-ray)	LRR	No	2-(ethoxymethyl)-1H-imidazo[4,5-c]quinolin-4-amine (C09), NAG, SO4, GOL	Yes
	3w3k	2013	2.30 (X-ray)	LRR	No	2-propyl[1,3]thiazolo[4,5-c]quinolin-4-amine (L07), NAG	Yes
	3w3l	2013	2.33 (X-ray)	LRR	No	1-[4-amino-2-(ethoxymethyl)-1H-imidazo[4,5-c]quinolin-1-yl]-2-methylpropan-2-ol (RX8), NAG, SO4	Yes
	3w3m	2013	2.70 (X-ray)	LRR	No	RX8, NAG, SO4	Yes
	3w3n	2013	2.10 (X-ray)	LRR	No	RX8, NAG, GOL	Yes
TLR9	8ar3	2023	Solution NMR	TM + cytoplasmic juxtamembrane regions	No	-	No
TLR10	2j67	2006	2.20 (X-ray)	TIR	No	-	No

Supplementary Table S2. Chemical structures of the best hits (small-molecule agonists and antagonists) from the reviewed research papers.

<table>
<tr><th colspan="4">AGONISTS</th></tr>
<tr><td colspan="2">Structures</td><td>Target</td><td>Ref</td></tr>
<tr><td>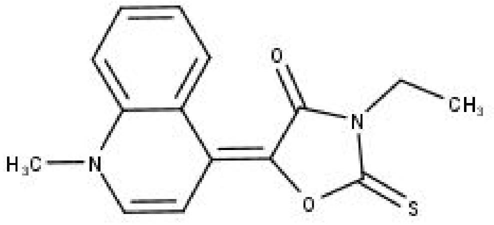
AG1</td><td>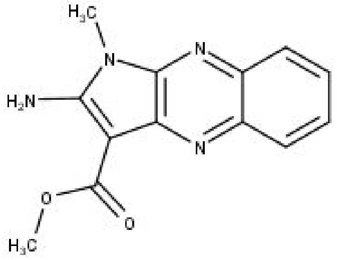
AG2</td><td rowspan="4">TLR2/1</td><td rowspan="4">38</td></tr>
<tr><td>Interacting amino acids:
hydrogen bonds: Thr361*, Asp362*, Thr363*, Lys385*, Leu371, Lys347
*indicates a TLR1 monomer</td><td>Interacting amino acids:
Thr363*, His318, Lys347
*indicates a TLR1 monomer</td></tr>
<tr><td>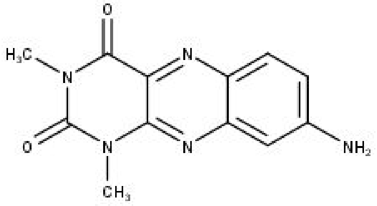
AG3</td><td>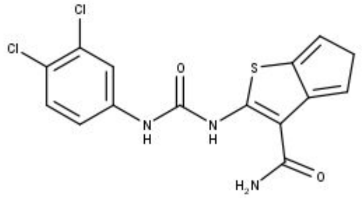
AG4</td></tr>
<tr><td>Interacting amino acids:</td><td>Interacting amino acids:</td></tr>
</table>

Asp362*, Thr363*, Lys385*, His318, Lys347<br>*indicates a TLR1 monomer	Thr361*, Glu386*, Asp286, His318, Lys347, Leu371<br>*indicates a TLR1 monomer		
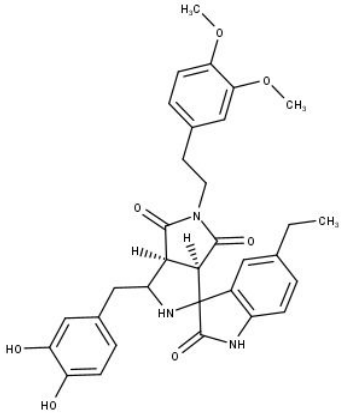<br>ZINC6662436 (SMU127)	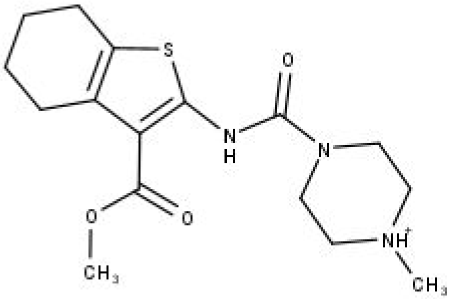<br>SMU-C13	TLR2/1	43,44
Interacting amino acids:<br>Phe312*, Gly313*, Gln316*, Gly313, Phe349<br>*indicates a TLR1 monomer	Interacting amino acids:<br>Phe312*, Gly313*, Phe314*, Pro315*, Gln316*, Lue324, Phe325, Tyr326, Asp327, Phe349, Leu350, Pro352<br>*indicates a TLR1 monomer		

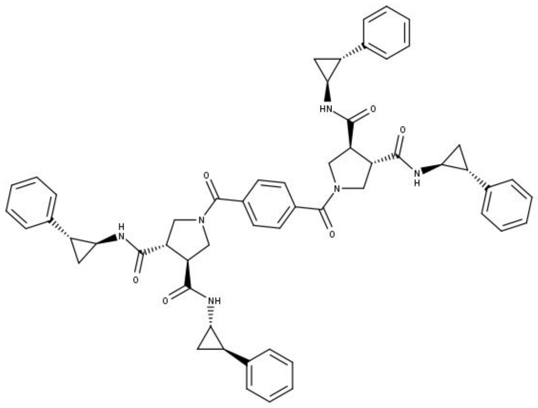<br>Diprovocim		TLR2/1	50,51
Interacting amino acids:<br>Trp258*, Leu287*, Ser309*, Val311*, Phe312*, Phe314*, Ser317*, Leu317, Ile319*, Met338*, Val339*, His430*<br>Leu317,Val348, Leu350, Phe394 (Diprovocim A)<br>Ile319, Tyr323, Phe325, Tyr326, Leu328 (Diprovocim B)<br>*indicates a TLR1 monomer			
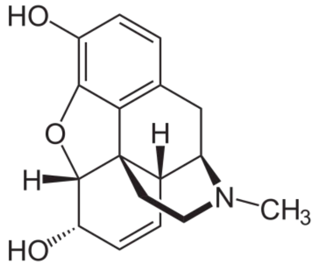<br>Morphine	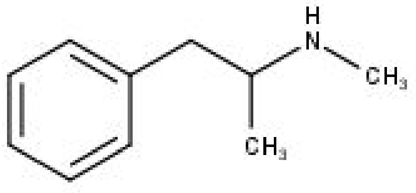<br>Methamphetamine	TLR4	55-57
Interacting amino acids from MD2:	Interacting amino acids from MD2:		

Val48, Ile63, Phe76, Phe147	Phe414*, Ser415*, Ile80^, Ile124^, Phe126^, Tyr131^<br>*indicates the TLR4 monomer<br>^indicates MD2 protein		
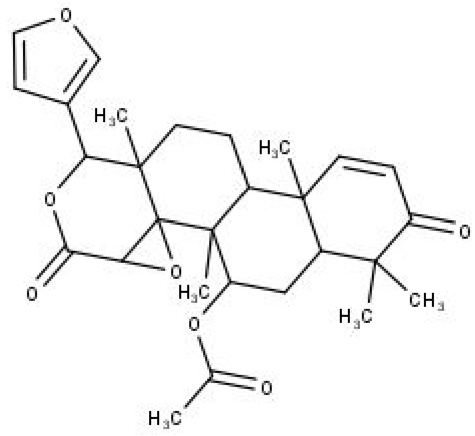<br>Gedunin		TLR2<br>TLR3<br>TLR4	64
Interacting amino acids from TLR2:<br>Lys698, Ala732, Leu762, Ala781, Ile733, Phe725, Leu734<br>Interacting amino acids from TLR3:<br>Ans257, Tyr283, Tyr307, Cys651, Leu640, Phe647, Ser653, Val658, Trp660<br>Interacting amino acids from caspase-1:<br>Arg383, His342, Met345, Val348			

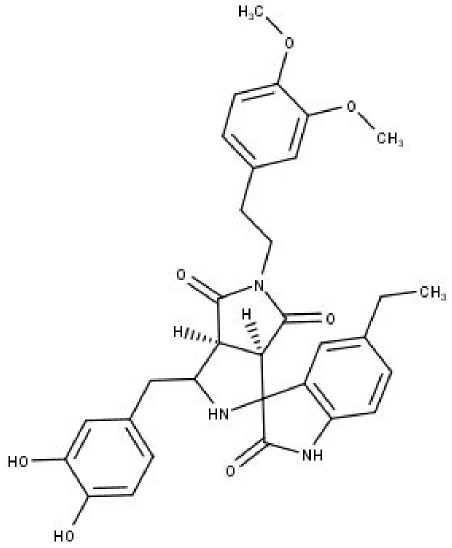<br>STOCK1N-65837		TLR7	66
Residues involved in hydrogen bonding from TLR7:<br>Lys509*, Gly437^<br>*indicates first TLR7 monomer<br>^indicates second TLR7 monomer			
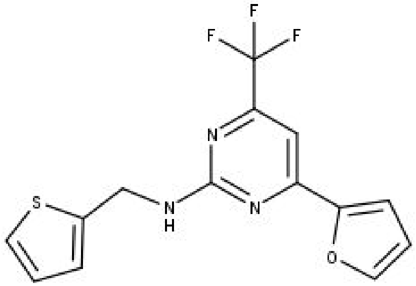<br>C5	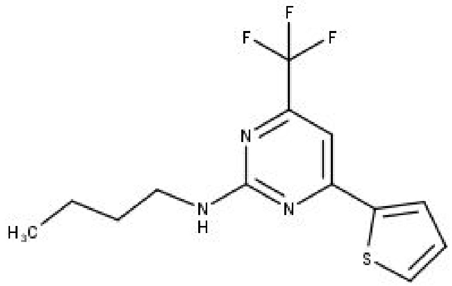<br>C11	TLR8	67

Interacting amino acids from TLR8<br>Phe494*, Phe495*, Ala518*, Gln519*, Val520*, Tyr567*<br>Phe261^, Tyr348^, Lys350^, Gly351^, Val378^, Phe405^<br>*indicates first TLR8 monomer<br>^indicates second TLR8 monomer			
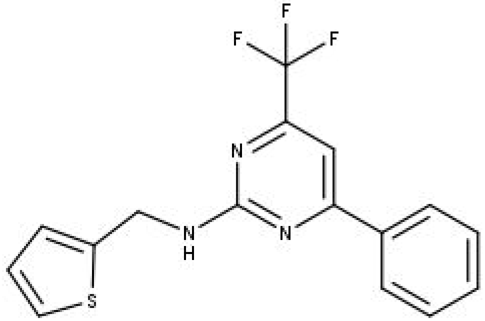<br>C14			
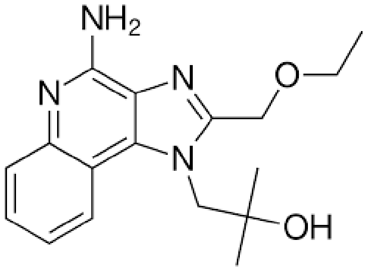<br>Resiquimod	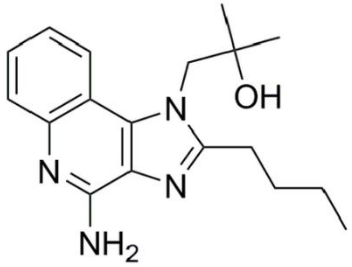<br>Hybrid-2	TLR7<br>TLR8	68
Interacting amino acids from TLR7:<br>Phe351*, Tyr356*, Phe408*, Asp555^, Leu557^, Ile585^, Thr586^	Interacting amino acids from TLR7:<br>Tyr346*, Phe351*, Val381*, Phe408*, Asp555^, Leu557^, Ile585^, Thr586^		

indicates first TLR7 monomer<br>^indicates second TLR7 monomer<br>Interacting amino acids from TLR8:<br>Tyr348, Tyr353*, Phe405*, Asp543^, Val573^, Thr574^<br>*indicates first TLR8 monomer<br>^indicates second TLR8 monomer	*indicates first TLR7 monomer<br>^indicates second TLR7 monomer<br>Interacting amino acids from TLR8:<br>Tyr348*, Tyr353*, Phe405*, Asp543^, Val573^, Thr574^<br>*indicates first TLR8 monomer<br>^indicates second TLR8 monomer		
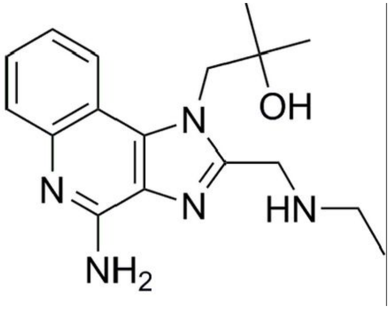<br>Gardiquimod			
Interacting amino acids from TLR7:<br>Phe351*, Tyr356*, Phe408*, Asp555^, Leu557^, Thr586^<br>*indicates first TLR7 monomer<br>^indicates second TLR7 monomer<br>Interacting amino acids from TLR8:<br>Tyr348*, Tyr353*, Phe405*, Asp543^, Asp545^<br>*indicates first TLR7 monomer<br>^indicates second TLR7 monomer			

**ANTAGONISTS**			
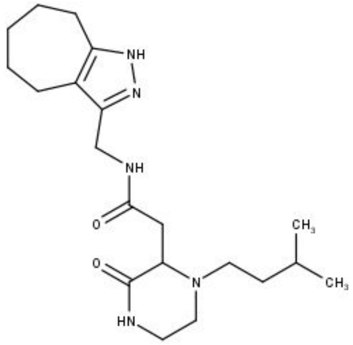<br>C11	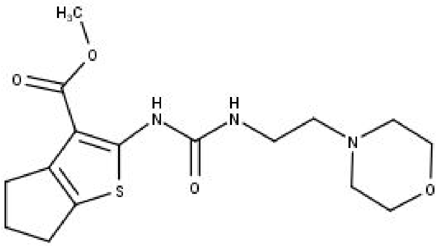<br>C13	TLR2/1	41
Interacting amino acids:<br>Asp310*, Val311*, Phe312*, Phe314*, Pro315*, Gln316*, Ile319, Phe322, Tyr323, Leu324, Phe325, Tyr326, Asp327, Leu328, Val343, Val348, Phe349, Leu350, Pro352, Ser363<br>*indicates a TLR1 monomer	Interacting amino acids:<br>Phe312*, Gly313*, Phe314*, Pro315*, Gln316*, Ile319, Phe322, Tyr323, Leu324, Phe325, Tyr326, Asp327, Leu328, Val343, Lys347, Val348, Phe349, Leu350<br>*indicates a TLR1 monomer		
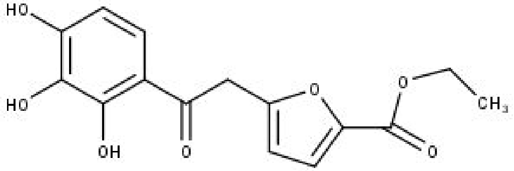<br>MMG-11		TLR2/1<br>TLR2/6	47
Interacting amino acids from TLR2:			

<table>
<tr><td>Leu266, Leu289, Phe284, Phe295, Ile314, Phe325, Leu328, Ser346, Lys347, Phe349, Leu350</td><td></td><td></td><td></td></tr>
<tr><td>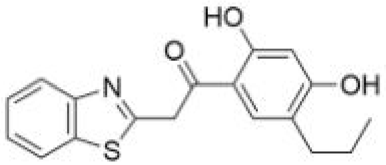
Cmp8</td><td></td><td rowspan="4">TLR2
TLR8
TLR7</td><td rowspan="4">48</td></tr>
<tr><td>Interacting amino acids:
Ile312*, Phe322*, Ser346*, Lys347*, Phe349*
Tyr348**, Ile349**, Gly351**, Val520**
*indicates a TLR2 monomer
** indicates a TLR8 monomer</td><td></td></tr>
<tr><td>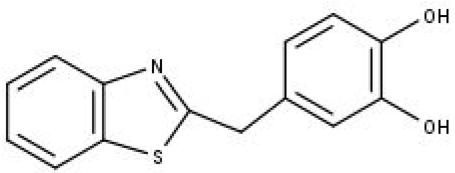
Cmp24</td><td></td></tr>
<tr><td>Interacting amino acids:
Ile312*, Phe322*, Ser346*, Lys347*, Phe349*
Tyr348**, Ile349**, Gly351**, Val520**
*indicates a TLR2 monomer
** indicates a TLR8 monomer</td><td></td></tr>
</table>

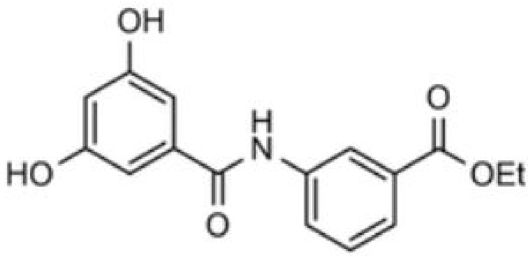<br>C6		TLR2/1<br>TLR2/6	49
Interacting amino acids from TLR2:<br>Leu226, Ile261, Phe284, Leu289, Leu314, Leu317, Phe325, Leu328, Val343, Ser346, Lys347, Phe349, Leu350			
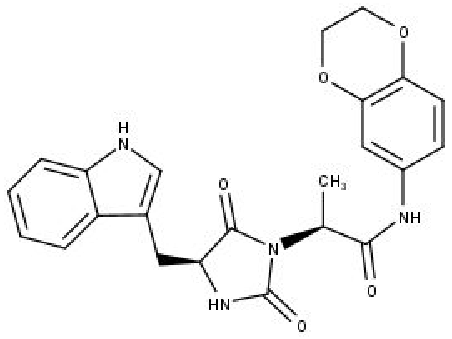<br>C11	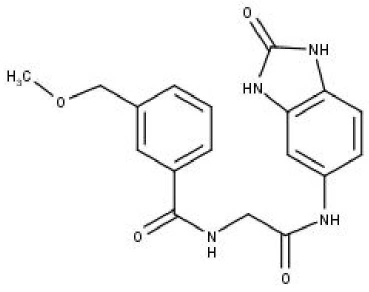<br>C15	TLR4	52
Interacting amino acids:<br>Arg264*, Leu61^, Ile63^, Pe76^, Val93^, Ile94^, Cys95^, Arg96^, Asp100^, Asp101^, Tyr102^, Phe104^, Ile117^<br>*indicates the TLR4 monomer<br>^indicates MD2 protein	Interacting amino acids:<br>Gln436*, Glu439*, Phe440*, Ile80^, Val82^, Leu87^, Arg90^, Phe126^, Ser126^, Tyr131^<br>*indicates the TLR4 monomer<br>^indicates MD2 protein		

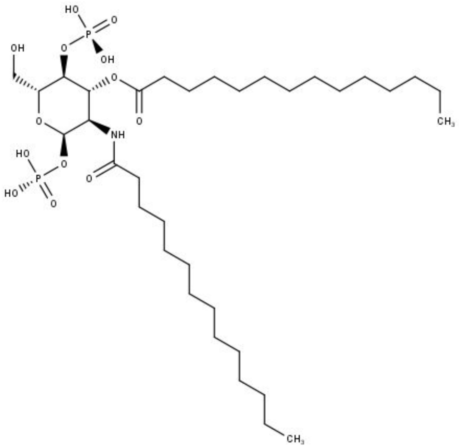<br>FP7(C14)	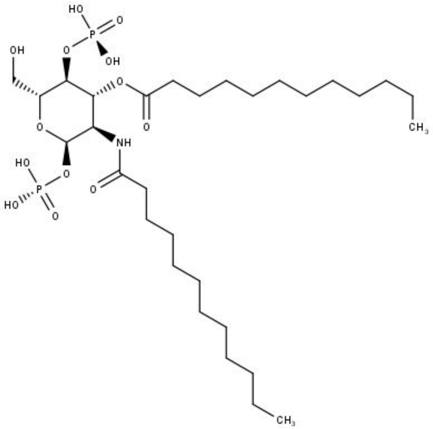<br>FP12(C12)	TLR4	53,54
Interacting amino acids from MD2:<br>Val24, Ala30, Ile32, Ile44, Ile46, Val48, Ile52, Leu54, Leu61, Ile63, Tyr65, Phe76, Leu78, Ile80, Arg90, Tyr102, Phe104, Val113, Ile117, Phe119, Phe121, Ser120, Ile124, Tyr131, Val135, Phe147, Leu149, Phe151, Ile153			

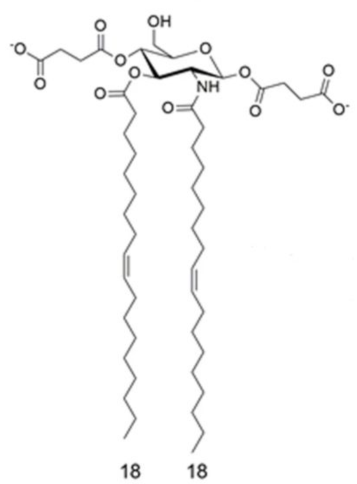 FP13	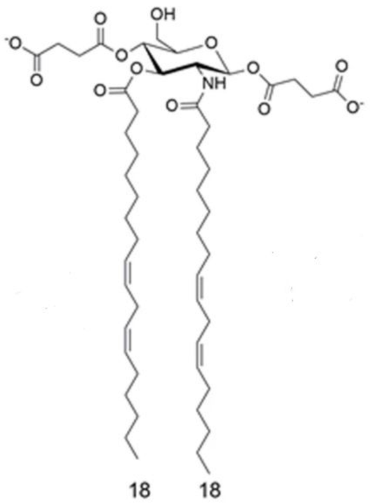 FP14		
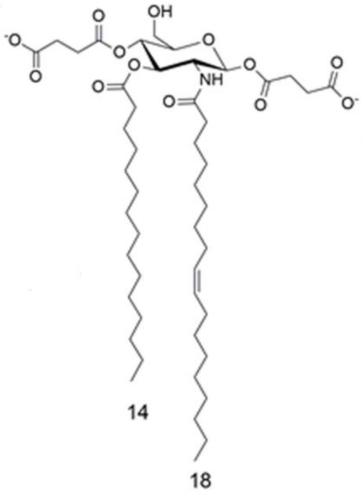 FP15	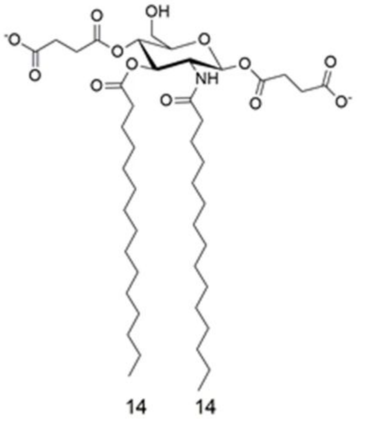 FP17		

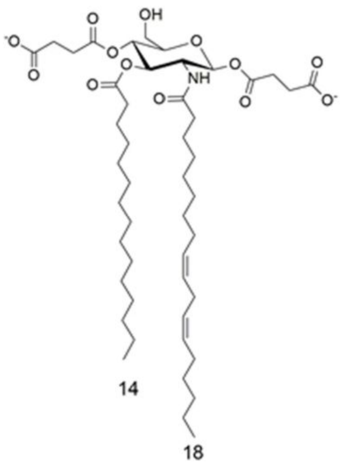<br>FP16			
Interacting amino acids from MD2:<br>Ile44, Ile46, Glu92, Arg96, Tyr102, Phe121, Phe147, Phe151			
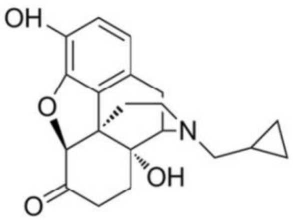<br>(+)-naltrexone	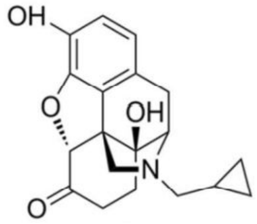<br>(-)-naltrexone	TLR4	58, 59, 60
Interacting amino acids from MD2:<br>Phe76, Glu92, Phe119, Phe121, Ile52, Phe151			

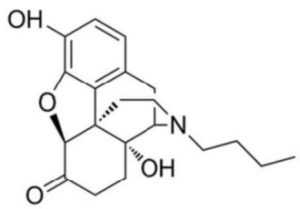 (+)-N-butylnoroxymorphone	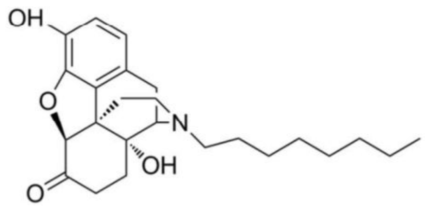 (+)-N-octylnoroxymorphone		
Interacting amino acids from MD2:<br>Ile52, Phe76, Leu94, Glu92, Phe119, Phe121, Phe151	Interacting amino acids from MD2:<br>Phe76, Arg90, Glu92, Leu94, Pro118, Phe119, Ser120		
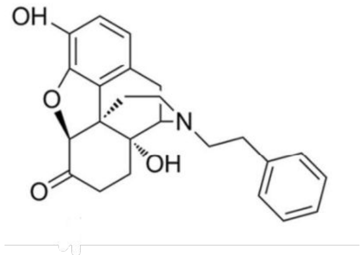 (+)-N-phenethylnoroxymorphone	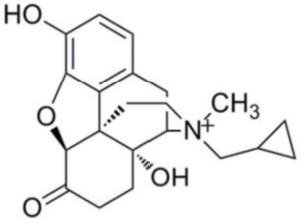 (+)-N-methylnaltrexone		
Interacting amino acids from MD2:<br>Ile52, Phe76, Leu94, Phe119, Phe147, Cys148, Ile149,<br>Phe151, Val161			

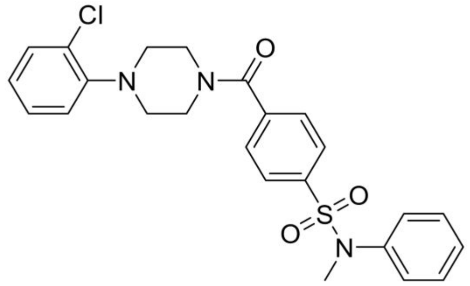<br>ID-5382 (B)	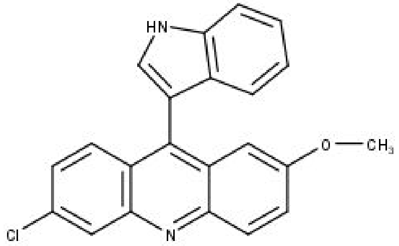<br>MS21 (F)	TLR4	62
Interacting amino acids from MD2:<br>: Ile46, Leu63, Leu78, Arg90, Phe121, Lys122, Ile124, Val135, Phe151	Interacting amino acids from MD2:<br>Leu61, Phe76, Val135, Phe147, Phe151		
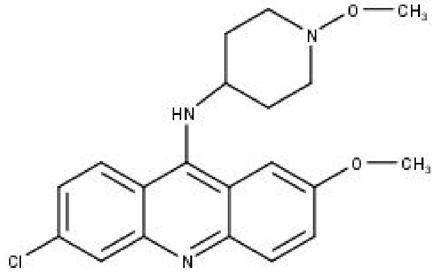<br>MS32 (H)	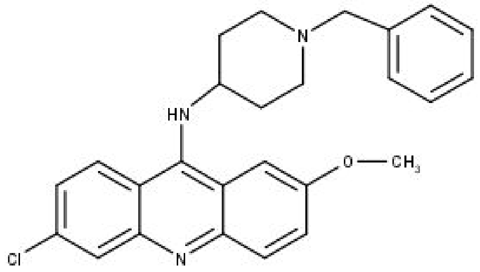<br>MS35 (I)		
Interacting amino acids from MD2:<br>Val48, Leu61, Ile63, Phe76, Val135, Phe151			

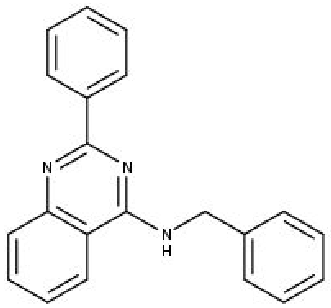<br><br>MS45 (J)	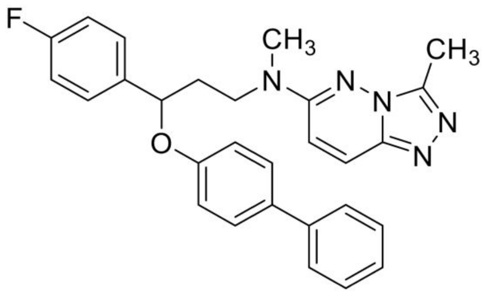<br><br>PM1090 (X)		
	Interacting amino acids from MD2:<br>Val48, Leu54, Leu61, Ile63, Phe76, Phe126, Val135, Phe147, Phe151, Val153		
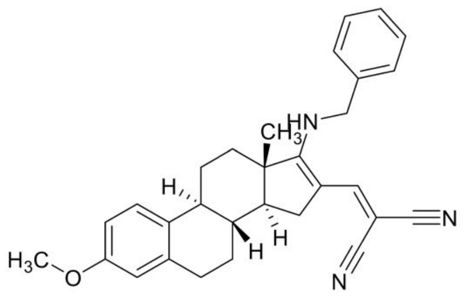<br><br>PM1200 (Z)	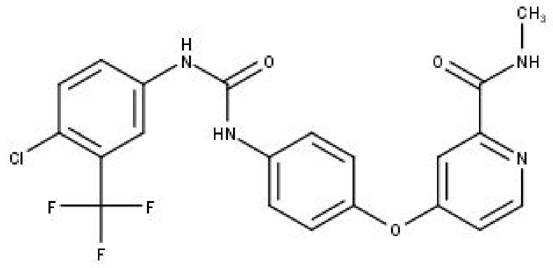<br><br>Sorafenib (M4)		
